# Long working hours and cancer risk: a multi-cohort study

**DOI:** 10.1038/bjc.2016.9

**Published:** 2016-02-18

**Authors:** Katriina Heikkila, Solja T Nyberg, Ida E H Madsen, Ernest de Vroome, Lars Alfredsson, Jacob J Bjorner, Marianne Borritz, Hermann Burr, Raimund Erbel, Jane E Ferrie, Eleonor I Fransson, Goedele A Geuskens, Wendela E Hooftman, Irene L Houtman, Karl-Heinz Jöckel, Anders Knutsson, Markku Koskenvuo, Thorsten Lunau, Martin L Nielsen, Maria Nordin, Tuula Oksanen, Jan H Pejtersen, Jaana Pentti, Martin J Shipley, Andrew Steptoe, Sakari B Suominen, Töres Theorell, Jussi Vahtera, Peter J M Westerholm, Hugo Westerlund, Nico Dragano, Reiner Rugulies, Ichiro Kawachi, G David Batty, Archana Singh-Manoux, Marianna Virtanen, Mika Kivimäki, for the IPD-Work Consortium

**Affiliations:** 1 Department of Health Services Research and Policy, London School of Hygiene and Tropical Medicine, London WC1H 9SH, UK; 2 Finnish Institute of Occupational Health, 33100 Tampere and 205200 Turku, Helsinki 0250, Finland; 3 National Research Centre for the Working Environment, Copenhagen DK-2100, Denmark; 4 TNO, Leiden 2316 ZL, The Netherlands; 5 Centre for Occupational and Environmental Medicine, Stockholm County Council, Sweden; 6 Institute of Environmental Medicine, Karolinska Institutet, Stockholm 171 77, Sweden; 7 Køge Hospital, Køge 4600, Denmark; 8 Federal Institute for Occupational Safety and Health, Berlin 10317, Germany; 9 Department of Cardiology, West-German Heart Center Essen, University Duisburg-Essen, Essen 45122, Germany; 10 Department of Epidemiology and Public Health, University College London, London WC1E 6BT, UK; 11 School of Social and Community Medicine, University of Bristol, Bristol BS8 2PS, UK; 12 School of Health and Welfare, Jönköping University, SE-551 11 Jönköping, Sweden; 13 Stress Research Institute, Stockholm University, Stockholm SE-106 91, Sweden; 14 Institute for Medical Informatics, Biometry and Epidemiology, Faculty of Medicine, University Duisburg-Essen, Essen 45122, Germany; 15 Department of Health Sciences, Mid Sweden University, Sundsvall 851 70, Sweden; 16 Department of Public Health, University of Helsinki, Helsinki 00140, Finland; 17 Institute for Medical Sociology, Medical Faculty, University of Düsseldorf, Düsseldorf 40225, Germany; 18 Unit of Social Medicine, Frederiksberg University Hospital, Fredriksberg 2000, Denmark; 19 Department of Psychology, Umeå University, Umeå 901 87, Sweden; 20 The Danish National Centre for Social Research, Copenhagen 1052, Denmark; 21 Department of Public Health, University of Turku, Turku 20014, Finland; 22 Folkhälsan Research Center, Helsinki 00290, Finland; 23 Nordic School of Public Health, Göteborg 426 71, Sweden; 24 Turku University Hospital, Turku 20521, Finland; 25 Occupational and Environmental Medicine, Uppsala University, Uppsala 751 85, Sweden; 26 Department of Public Health and Department of Psychology, University of Copenhagen, Copenhagen 2200, Denmark; 27 Department of Society, Human Development and Health, Harvard School of Public Health, Boston, Massachusetts 02115, USA; 28 Centre for Cognitive Ageing and Cognitive Epidemiology, University of Edinburgh, Edinburgh EH8 9JZ, UK; 29 Inserm U1018, Centre for Research in Epidemiology and Population Health, Villejuif 94807, France; 30 Clinicum, Faculty of Medicine, University of Helsinki, Helsinki FI-00014, Finland

**Keywords:** Breast cancer, colorectal cancer, lung cancer, prostate cancer, working hours

## Abstract

**Background:**

Working longer than the maximum recommended hours is associated with an increased risk of cardiovascular disease, but the relationship of excess working hours with incident cancer is unclear.

**Methods:**

This multi-cohort study examined the association between working hours and cancer risk in 116 462 men and women who were free of cancer at baseline. Incident cancers were ascertained from national cancer, hospitalisation and death registers; weekly working hours were self-reported.

**Results:**

During median follow-up of 10.8 years, 4371 participants developed cancer (*n* colorectal cancer: 393; *n* lung cancer: 247; *n* breast cancer: 833; and *n* prostate cancer: 534). We found no clear evidence for an association between working hours and the overall cancer risk. Working hours were also unrelated the risk of incident colorectal, lung or prostate cancers. Working ⩾55 h per week was associated with 1.60-fold (95% confidence interval 1.12–2.29) increase in female breast cancer risk independently of age, socioeconomic position, shift- and night-time work and lifestyle factors, but this observation may have been influenced by residual confounding from parity.

**Conclusions:**

Our findings suggest that working long hours is unrelated to the overall cancer risk or the risk of lung, colorectal or prostate cancers. The observed association with breast cancer would warrant further research.

Epidemiological research suggests that working long hours has a detrimental effect on health. Extended working hours have been reported as being associated with an increased incidence of coronary heart disease and stroke (Kang *et al*, 2012; Virtanen *et al*, 2012; Kivimaki *et al*, 2015a) pre-term delivery (van Melick *et al*, 2014) and, in manual occupations, type 2 diabetes (Kivimaki *et al*, 2015b), as well as a high prevalence of anxiety, depression, sleeping difficulties and accidental injuries at work. (Dembe *et al*, 2005; Bannai and Tamakoshi, 2014). The relationship between long working hours and cancer, however, is unclear.

Long working hours could impact on cancer risk via their association with lifestyle-related exposures. Observational evidence suggests that working longer than recommended hours is linked to many behavioural cancer risk factors, such as excessive alcohol intake (Virtanen *et al*, 2015) and physical inactivity (Kirk and Rhodes, 2011; Angrave *et al*, 2015), possibly because individuals feel that they lack time to exercise because they spend extensive time at work (Escoto *et al*, 2012). As far as we are aware, the association between long working hours and incident cancer has been examined in only one previous investigation, which had inconclusive findings: in that prospective cohort study the association between working 45 h or longer per week and breast cancer was imprecisely estimated (hazard ratio (HR): 0.93, 95% confidence interval (CI): 0.54, 1.58) and no other cancer outcomes were examined (Nielsen *et al*, 2008).

To address this evidence gap, we examined the relationship between weekly working hours and the overall incident cancer as well as incident colorectal, lung, breast and prostate cancers using individual participant data from 116 000 men and women from 12 prospective cohort studies from six European countries.

## Materials and Methods

### Studies

The 12 studies in our analyses were conducted between 1992 and 2004 in Denmark, Finland, Germany, Sweden, The Netherlands and UK. All were a part of the Individual-Participant-Data Meta-analysis of Working Populations (IPD-Work) Consortium, a collaborative research effort to investigate the health impact of work-related exposures (Kivimaki *et al*, 2012). Details of each study's design, recruitment of participants, data collection and ethics committee approval are provided in Supplementary eAppendix 1.

### Participants

Our analyses were based on 116 462 men and women who were working and free of cancer at study baseline, whose records were linked to register-based information on incident cancers and who had complete data available on covariates (Supplementary eAppendix 1 and Supplementary Table S1).

### Exposures and outcomes

Weekly working hours were ascertained from baseline self-report questions on usual weekly working hours and defined as the total number of hours in the main job and any secondary jobs (Supplementary eAppendix 2 and Supplementary Table S2).

Cancer events were identified from national cancer, hospitalisation and death registers in all studies apart from one (for details, see Supplementary eAppendix 2). The date of the cancer event was defined as the date of diagnosis or hospital admission due to cancer, whichever was earlier. Cancer cases were categorised according to the type and time of diagnosis of their first cancer. We excluded individuals whose first cancer record came from their death certificate (*n*=10), as the date of diagnosis for these cancers was uncertain. Codes for the incident cancer events were harmonised using ICD-10 (International Classification of Diseases, version 10) as any cancer (ICD-10 codes C00-C97), colorectal (C18-C20), lung (C34), female breast (C50) and prostate (C61) cancers.

### Potential confounders and mediators

Details of the selection and ascertainment of the covariates included in our models are provided in Supplementary eAppendix 2. Briefly, potential confounders were age, sex, socioeconomic position, shift work and night-time work. Potential mediators were smoking, alcohol intake and body mass index (BMI). All covariates, measured at baseline, were harmonised across the studies as reported previously (Heikkila *et al*, 2012; Heikkilä *et al*, 2012; Nyberg *et al*, 2012, 2014).

### Statistical analysis

Weekly working hours were analysed as a categorical exposure: <35 h, 35–40 h (reference category: standard working hours for the majority of the workforce in Europe), 41–48 h (the upper limit for the European Union Working Time Directive), 49–54 h and ⩾55 h. Incident cancers (any cancer, colorectal, lung, female breast and prostate cancers) were analysed as binary outcomes. Each participant was followed-up from the date of their baseline assessment to the earliest of the following: incident cancer, death or the end of the registry follow-up. We modelled the associations between working hours and each cancer outcome in each study using Cox proportional hazards regression with the participant's age (i.e., time since birth) as the time scale in the model. Study-specific results were combined using random effects meta-analyses. All statistical analyses were conducted using Stata MP 13 (Stata Corporation, College Station, TX, USA) bar the study-specific analyses in the Danish studies, which were conducted using SAS 9.3 (SAS Institute Inc., Cary, NC, USA) and POLS, which were conducted using SPSS 20.0 (SPSS Inc., Chicago, IL, USA).

## Results

The characteristics of the 116 462 participants are summarised in Table 1. Overall, these men and women were aged 15–73 at baseline and the majority worked a standard 35–40 h per week, with the study-specific proportions varying from 31 to 71%. During a follow-up ranging from 4 to 22 years (median of study-specific medians: 10.8), 4371 individuals were diagnosed with cancer. Of these, 393 men and women had colorectal cancer and 247 had lung cancer; 833 women developed breast cancer and 534 men prostate cancer.

**Figure 1:**
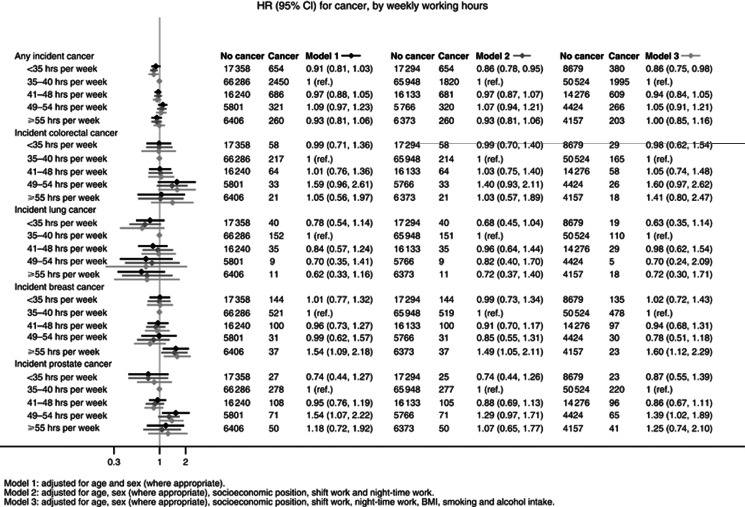
Associations of weekly working hours with incident cancer.

The associations between weekly working hours and incident cancers are shown in Figure 1. The study-specific estimates are provided in Supplementary eAppendices 3–7. We observed no association between longer than recommended weekly working hours and overall cancer risk, although working <35 h per week was associated with a slightly reduced average risk of any incident cancer (multivariable-adjusted random effects HR: 0.86, 95% CI: 0.76, 0.98). Our meta-analyses provided no clear evidence for an association between weekly working hours and the risk of colorectal or lung cancers. Working hours were also generally unrelated to incident prostate cancer, though the risk was slightly elevated among men who worked 49–54 h per week (multivariable-adjusted HR: 1.39, 95% CI: 1.02, 1.89). There was negligible heterogeneity among the study-specific estimates for these cancer outcomes. Generally, adjustment for work-related factors (socioeconomic position, night-time work and shift work) or lifestyle factors (BMI, smoking or alcohol intake) had little impact on the estimates.

Working 55 h or longer was associated with an increased risk of female breast cancer in the age-adjusted analyses (HR: 1.54, 95% CI: 1.09, 2.18). This association remained after additional adjustment for socioeconomic position; night-time work, shift work (HR: 1.49, 95% CI: 1.05, 2.11) and BMI; smoking; and alcohol intake (HR: 1.60, 95% CI: 1.12, 2.29). The study-specific estimates were similar to one another in direction and magnitude (*I*^2^: <0%).

## Discussion

In our study of over 116 000 European men and women and up to 4371 incident cancer cases, we found no evidence for an association between long weekly working hours and the overall cancer incidence, although those working <35 h per week had a slightly reduced risk. No evidence was observed for an association between weekly working hours and the risks of colorectal, lung or prostate cancers. Working 55 h or longer per week was associated with an increased breast cancer risk (multivariable-adjusted random effects HR: 1.60, 95% CI: 1.12, 2.29). Overall, there was little heterogeneity among the study-specific association estimates and adjustment for work characteristics, socioeconomic position, obesity and lifestyle factors did not markedly change these.

To our knowledge, ours is the largest investigation of this topic to-date and the first to examine the association of working hours with the overall cancer risk as well as the specific risks of common cancers. In the IPD-Work Consortium we have previously reported associations of work-related stress exposures with cardiovascular disease outcomes but not with incident cancers (Kivimaki *et al*, 2012; Heikkila *et al*, 2013; Nyberg *et al*, 2013; Nyberg *et al*, 2014; Fransson *et al*, 2015; Kivimaki *et al*, 2015a; Kivimaki *et al*, 2015b), findings that the current observations seem to support. Our findings are also in keeping with the only previous study of this topic. Working 45 h or longer per week was reported being unrelated to breast cancer risk among female Danish nurses aged 44 years and over (HR: 0.93, 95% CI: 0.54, 1.58) (Nielsen *et al*, 2008). The categorisation of weekly working hours as well as the reference category in this study were different from ours, and the estimates thus not directly comparable, but the previously published null-association is compatible with our estimates for similar exposure categories (41–48 h per week, HR: 0.94, 95% CI: 0.68, 1.31) and 49–54 h per week, HR: 0.78, 95% CI: 0.51, 1.18). As no other cancer outcomes were examined in the Danish Nurse Cohort study, we were unable to gauge the compatibility of the rest of our findings with previous research.

The association of working 55 h or longer per week with incident breast cancer should be interpreted with caution: no trend in risk was observed across the working-hour categories and this association could thus have been observed by chance or it could relate to the residual confounding. The observed association between these extensively long working hours and incident breast cancer was not markedly influenced by adjustment for lifestyle factors, shift work or night-time work, the latter of which has been suggested to increase breast cancer risk by disrupting the body's circadian rhythms and altering the nocturnal melatonin production, thus impacting on the development of hormone-related breast cancers. However, the evidence for the relationship between night-time work and breast cancer has been recently summarised in systematic reviews and meta-analyses, which showed that the associations reported in case–control studies were not corroborated by prospective evidence. (Ijaz *et al*, 2013; Jia *et al*, 2013; Kamdar *et al*, 2013; Wang *et al*, 2013). One important factor that could have a role in the relationship between working hours and breast cancer, and would merit further research, is parity (Ewertz *et al*, 1990; Collaborative Group on Hormonal Factors in Breast Cancer, 2002): it could be a confounder or a mediator, as women who work long hours may have fewer children because of childcare demands or cost, or women with children may restrict their working hours. Other potentially relevant exposures include age at first birth, menopausal status, use of hormone replacement therapy and sedentary behaviour at work (Schmid and Leitzmann, 2014). However, as we had no harmonised data on these factors, we were unable to investigate them further.

It is unclear what the slightly reduced overall cancer risk among men and women working fewer than 35 h per week relates to (multivariable-adjusted HR: 0.86, 95% CI: 0.75, 0.98). As the association between working hours and incident prostate cancer was not consistent across the exposure categories, we suspect that the slightly elevated risk observed in men who worked 49–54 h per week is a chance finding.

As our investigation was based on previously unpublished data, the findings presented here have not been influenced by publication bias. Our analyses were based on a relatively large number of participants from several countries, and with occupations ranging from manual labour to managerial positions, making our findings widely generalisable to the working populations in the Northern and Western Europe. However, at the same time this limits the generalisability of our observations to other continents or low-income countries.

In conclusion, our findings suggest that long working hours are unlikely to be associated with the overall cancer risk or the specific risks of colorectal, lung or prostate cancers. The observed association between very long working hours and increased breast cancer risk should be interpreted cautiously and would warrant further research.

## Figures and Tables

**Table 1:** Participant characteristics

							**Working hours**		**Incident cancer**	
**Study**	**Baseline Year**	**Country**	***N*** **Participants**^a^	**Follow-up (years) Median**	***N* (%) Men**	**Age Mean (s.d.)**	**Category**	***N* (%)**	**Type**	***N***
WOLF Stockholm	1992	Sweden	5363	14.8	3117 (58.1)	41.3 (11.0)	<35 35–40 41–48 49–54 ⩾55	281 (6.2) 2397 (52.7) 1666 (36.6) 152 (3.3) 55 (1.2)	Any Colorectal Lung Breast Prostate	468 51 28 61 83
Whitehall II	1992–1993	UK	7341	22.6	5096 (69.4)	48.8 (5.7)	<35 35–40 41–48 49–54 ⩾55	229 (3.1) 3865 (52.7) 1458 (19.9) 1057 (14.4) 732 (10.0)	Any Colorectal Lung Breast Prostate	953 96 38 146 175
WOLF Norrland	1996	Sweden	4551	11.8	3838 (84.3)	43.9 (10.2)	<35 35–40 41–48 49–54 ⩾55	527 (9.8) 2614 (48.7) 1611 (30.0) 385 (7.2) 226 (4.2)	Any Colorectal Lung Breast Prostate	255 32 18 17 66
IPAW	1996–1997	Denmark	1989	14.0	661 (33.2)	41.1 (10.4)	<35 35–40 41–48 49–54 ⩾55	648 (32.6) 1244 (62.5) 77 (3.9) 14 (0.7) 6 (0.3)	Any Colorectal Lung Breast Prostate	142 12 18 38 8
COPSOQ-I	1997	Denmark	1788	13.1	928 (51.9)	40.5 (10.6)	<35 35–40 41–48 49–54 ⩾55	342 (19.1) 974 (54.5) 249 (13.9) 113 (6.3) 110 (6.2)	Any Colorectal Lung Breast Prostate	105 11 7 24 4
HeSSup	1998	Finland	15 888	8.0	7151 (45.0)	39.5 (10.2)	<35 35–40 41–48 49–54 ⩾55	1882 (11.9) 8511 (53.6) 2912 (18.3) 1176 (7.4) 1407 (8.9)	Any Colorectal Lung Breast Prostate	401 25 9 109 39
PUMA	1999	Denmark	1740	11.1	307 (17.6)	42.6 (10.1)	<35 35–40 41–48 49–54 ⩾55	557 (32.0) 1013 (58.2) 120 (6.9) 33 (1.9) 17 (1.0)	Any Colorectal Lung Breast Prostate	105 12 10 30 6
DWECS	2000	Denmark	5439	10.5	2924 (53.8)	41.6 (11.0)	<35 35–40 41–48 49–54 ⩾55	884 (16.3) 3002 (55.2) 788 (14.5) 330 (6.1) 435 (8.0)	Any Colorectal Lung Breast Prostate	227 21 19 49 23
FPS	2000	Finland	42 794	4.5	8528 (19.9)	44.4 (9.4)	<35 35–40 41–48 49–54 ⩾55	3413 (8.0) 30 475 (71.2) 6108 (14.3) 1440 (3.4) 1358 (3.2)	Any Colorectal Lung Breast Prostate	860 37 27 310 44
HNR	2000	Germany	1833	9.2	1074 (58.6)	53.5 (5.1)	<35 35–40 41–48 49–54 ⩾55	473 (25.8) 559 (30.5) 289 (15.8) 206 (11.2) 306 (16.7)	Any Colorectal Lung Breast Prostate	150 8 17 21 25
POLS	1997–2002	Netherlands	24 417	9.9	14 382 (58.9)	38 (11.1)	<35 35–40 41–48 49–54 ⩾55	8253 (33.8) 12 331 (50.5) 1001 (4.1) 1001 (4.1) 1831 (7.5)	Any Colorectal Lung Breast Prostate	624 79 49 10 58
COPSOQ-II	2004	Denmark	3319	6.0	1585 (47.7)	42.6 (10.2)	<35 35–40 41–48 49–54 ⩾55	528 (15.9) 1748 (52.7) 658 (19.8) 212 (6.4) 173 (5.2)	Any Colorectal Lung Breast Prostate	81 9 7 18 3

Abbreviations: COPSOQ-I=Copenhagen Psychosocial Questionnaire I; COPSOQ-II=Copenhagen Psychosocial Questionnaire II; DWECS=Danish Work Environment Cohort Study; FPS=Finnish Public Sector Study; HeSSup=Health and Social Support Study; HNR=Heinz-Nixdorf Recall Study; IPAW=Intervention Project on Absence and Well-being; POLS=Permanent Onderzoek Leefsituatie; WOLF=Work, Lipids and Fibrinogen.

a With complete data on weekly working hours, cancer outcomes, age and sex, and free of cancer at study baseline and within the first year of follow-up.
