# Supplementary material for: Long working hours and cancer risk: a multi-cohort study
Source: Br J Cancer. 2016 Feb 18;114(7):813–8. doi: 10.1038/bjc.2016.9 (PMC4984872; doi:10.1038/bjc.2016.9)

eAppendix 1. Details of studies

Details of the design and data collection in the studies included in our analyses are presented below. Participants were eligible for the analyses if they were working and had data available on weekly working hours at baseline.

Copenhagen Psychosocial Questionnaire version I (COPSOQ-I)

The COPSOQ-I is a prospective cohort study of a random sample of Danish residents selected from the Danish population register. The participants were aged 20-60 years of age and were in paid employment at the study baseline in 1997. A baseline questionnaire and an invitation to take part was posted to 4 000 people and 2 454 individuals agreed to participate. [1] In Denmark, questionnaire- and register-based studies do not require approval from the Danish National Committee on Biomedical Research Ethics (Den Centrale Videnskabetiske komité). COPSOQ-I was approved by and registered with the Danish Data protection agency (registration number: 2008 - 54 - 0553).

Copenhagen Psychosocial Questionnaire version II (COPSOQ-II)

COPSOQ-II was carried out in 2004-2005. It included a follow up of respondents from COPSOQ I and also a representative sample of Danish residents aged 20-60 at study baseline. The questionnaire was sent to 8 000 individuals from the random sample and 4 732 individuals responded, returning the questionnaire by post or via the internet. [2] In Denmark questionnaire- and register-based studies do not require ethics committee approval. COPSOQ-II was approved by and registered with the Danish Data protection agency (registration number: 2004-54-1493).

Danish Work Environment Cohort Study (DWECS)

DWECS is a split panel survey of working age Danish people. The cohort was established in 1990, when a simple random sample of men and women, aged 18-59, was drawn from the Danish population register. The participants have been followed up at five year intervals and data from the year 2000 were used for the IPD-Work. That year 11 437 individuals were invited to participate and 8 583 agreed to do so. [3, 4] In Denmark questionnaire- and register-based studies do not require ethics committee approval. DWECS was approved by and registered with the Danish Data protection agency (registration number: 2007-54-0059).

Finnish Public Sector study (FPS)

The Finnish Public Sector study is a prospective cohort study comprising the entire public sector personnel of 10 towns or municipalities, and 21 hospitals in the same geographical areas. Participants, recruited from employers' records in 2000-2002, were individuals who were employed in the study organisations at the time of the questionnaire survey. [5] In 2000, 48 592 individuals aged 17 to 65 responded to the questionnaire. Ethical approval was obtained from the Helsinki and Uusimaa hospital district ethics committee.

Health and Social Support (HeSSup)

The Health and Social Support (HeSSup) study is a prospective cohort study of a stratified random sample of the Finnish population in the following four age groups: 20–24, 30–34, 40–44 and 50–54 years. The participants were identified from the Finnish population register and posted an invitation to participate in 1998. 25 898 individuals responded and returned the baseline questionnaire. [6] Turku University Central Hospital Ethics Committee approved the study.

Heinz Nixdorf Recall study (HNR)

Heinz Nixdorf Recall Study is a prospective population-based cohort study of individuals randomly selected from the mandatory lists of residence in the metropolitan Ruhr area in Germany. Details of the study methods have been described previously. [7, 8] Briefly, 4 814 participants aged 45-75 years were enrolled at study baseline in 2000-2003. Measures of work-related stress and comprehensive medical data were collected at the baseline examination. HNR was approved by the institutional local ethical committees and a quality management system according to European industrial norms (DIN EN ISO 9001:2000) was applied

Intervention Project on Absence and Well-being (IPAW)

IPAW is a 5-year psychosocial work environment intervention study including 22 intervention and 30 control work places in three organisations (a large pharmaceutical company, municipal technical services and municipal nursing homes) in Copenhagen, Denmark. [9, 10] The baseline questionnaire was posted to all the employees at the selected work-sites between 1996 and 1997. Interventions took place at 22 workplaces during 1996-98 at the organisational and interpersonal level. Of the 2 721 employees who worked at the IPAW sites, 2 068 men and women completed the baseline questionnaire. IPAW was approved by and registered with the Danish Data Protection Agency (registration number: 2000-54-0066).

Permanent Onderzoek Leefsituatie (POLS)

Permanent Onderzoek Leefsituatie (POLS) is a series of annual cross-sectional health and lifestyle surveys of Dutch men and women. [11] The participants are a representative sample of the Dutch population, drawn from the Municipal Population Register (Gemeentelijke Basis Administratie, GBA). Only those living in a private household were included. Most of the data collection is done using computer assisted personal interviewing. At study baseline in 1997-

2002, 59 441 men and women participated in the surveys. POLS was approved by the medical ethics committee of the Netherlands Organisation for Applied Scientific Research.

Burnout, Motivation and Job Satisfaction study (Danish acronym: PUMA)

Burnout, Motivation and Job Satisfaction study (Danish acronym: PUMA) is an intervention study of burn-out among employees in the human service sector. [12] Selection criteria for the participating organisations was that they had between 200 and 500 employees, that occupational groups within each organisation were willing to participate and that the organisations would commit to the entire five-year study period. Participants gave consent to having their national identity numbers collected and used in later record linkages to Danish hospitalisation and cause of death registries (Hospitalsindlæggelsesregisteret, Dødsårsagsregisteret. At study baseline in 1999-2000, 1 914 participants agreed to take part. PUMA was approved by the Scientific Ethical Committees (Videnskabsetisk Komiteer) in the counties in which the study was conducted and approved by and registered with the Danish Data Protection Agency (registration number: 2000-54-0048).

Whitehall II

Whitehall II is a prospective cohort study set up to investigate socioeconomic determinants of health. At study baseline in 1985-1988, 10 308 civil service employees aged 35-55 and working in 20 civil service departments in London were invited to participate in the study. [13] Data on weekly working hours were collected at study phase 3, in 1991-94, which was used as an analytical baseline in our investigation. The Whitehall II study protocol was approved by the University College London Medical School committee on the ethics of human research. Written informed consent was obtained at each data collection wave.

Work, Lipids, and Fibrinogen (WOLF) Stockholm and Norrland

WOLF Stockholm study is a prospective cohort study of 5 698 people (3 239 men and 2 459 women) aged 19–70 and working in companies in Stockholm county. [14] WOLF Norrland is a prospective cohort of 4 718 participants aged 19-65 working in companies in Jämtland and Västernorrland counties. [15] At study baseline the participants underwent a clinical examination and completed a set of health questionnaires. For WOLF Stockholm, the baseline assessment was undertaken at 20 occupational health units in 1992-95 and for WOLF Norrland at 13 occupational health service units in 1996-98. The Regional Research Ethics Board in Stockholm and the ethics committee at Karolinska Institutet, Stockholm, Sweden, approved the studies.

Table S1. Participants excluded from and included in the analyses

Study	N eligible participants[1]	Excluded							Included		
		**N cancer before study baseline**	**N cancer within the first 365 days of follow-up**	**First record of cancer in the death certificate**	**N incomplete follow-up**	**N missing data on age or sex**	**N missing data on age, sex, SEP, shift work or night-time work**	**N missing data on age, sex, SEP, shift work, night-time work, BMI, smoking or alcohol intake**	**N in model 1[2]**<br>N cancer-free<br>N any incident cancer	**N in model 2[3]**<br>N cancer-free<br>N any incident cancer	**N in model 3[4]**<br>N cancer-free<br>N any incident cancer
Work, Lipids and Fibrinogen (WOLF) Stockholm	5 567	183	18	2	1	0	83	232	**5 363**<br>4 895<br>468	**5 280**<br>4820<br>460	**5 131**<br>4 690<br>441
Whitehall II	7 399	49	3	6	0	0	4 [5]	529 [5]	**7 341**<br>6 388<br>953	**7 337**<br>6 386<br>951	**6 812**<br>5 937<br>875
Work, Lipids and Fibrinogen (WOLF) Norrland	4 671	105	14	1	0	0	31	138	**4 551**<br>4 296<br>255	**4 520**<br>4 267<br>253	**4 413**<br>4 169<br>245
Intervention Project on Absence and Well-being (IPAW)	2 031	34	8	0	0	0	0	105	**1 989**<br>1 847<br>142	**1 989**<br>1 847<br>142	**1 884**<br>1 754<br>130
Copenhagen Psychosocial Questionnaire I (COPSOQ-I)	1 820	24	3	0	5	0	7	74	**1 788**<br>1 683<br>105	**1 781**<br>1 677<br>104	**1 781**<br>1 677<br>104
Health and Social Support study (HeSSup)	16 242	317	36	1	0	0	20	1 408	**15 888**<br>15 487<br>401	**15 868**<br>15 467<br>401	**14 480**<br>14 104<br>376

Table S1, continued. Participants excluded from and included in the analyses

Study	N eligible participants[1]	Excluded							Included		
		**N cancer before study baseline**	**N cancer within the first 365 days of follow-up**	**First record of cancer in the death certificate**	**N incomplete follow-up**	**N missing data on age or sex**	**N missing data on age, sex, SEP, shift work or night-time work**	**N missing data on age, sex, SEP, shift work, night-time work, BMI, smoking or alcohol intake**	**N in model 1[2]**<br>N cancer-free<br>N any incident cancer	**N in model 2[3]**<br>N cancer-free<br>N any incident cancer	**N in model 3[4]**<br>N cancer-free<br>N any incident cancer
Burnout, Motivation and Job Satisfaction study (Danish acronym PUMA)	1 793	45	4	0	4	0	3	103	**1 740**<br>1 635<br>105	**1 737**<br>1 632<br>105	**1 634**<br>1 536<br>98
Danish Work Environment Cohort Study (DWECS)	5 592	106	16	0	31	0	0	69	**5 439**<br>5 212<br>227	**5 439**<br>5 212<br>227	**5 370**<br>5 148<br>222
Finnish Public Sector study (FPS)	44 518	1 534	190	0	0	0	447	3 280	**42 794**<br>41 934<br>860	**42 347**<br>41 495<br>852	**39 514**<br>38 731<br>783
Heinz-Nixdorf Recall study (HNR)	2 108	45	16	0	2	212	217	797	**1 833**<br>1 683<br>150	**1 828**<br>1 678<br>150	**1 248**<br>1 147<br>101
Permanent Onderzoek Leefsituatie (POLS)	24 547	52	39	0	0	39	4	15 569	**24 417**<br>23 793<br>624	**24 413**<br>23 789<br>624	**-** [6]<br>**-**<br>**-**
Copenhagen Psychosocial Questionnaire II (COPSOQ-II)	3 417	74	13	0	11	0	0	73	**3 319**<br>3 238<br>81	**3 319**<br>3 238<br>81	**3 246**<br>3 168<br>78

Footnotes to Table S1.

[1] Working at study baseline and data on weekly working hours available.
[2] With complete data on weekly working hours, cancer outcomes, age and sex.
[3] With complete data on weekly working hours, cancer outcomes, age, sex, SEP, night-time work and shift work.
[4] With complete data on weekly working hours, age, sex, SEP, night-time work, shift work, BMI, smoking and alcohol intake.
[5] In Whitehall II no-one worked shifts or at night, so these variables were not modelled as covariates in this study.
[6] POLS was excluded from the meta-analyses of multivariable-adjusted models due to difficulties with convergence in multivariable-adjusted models in this study.

References

1. Kristensen TS, Hannerz H, Hogh A, Borg V. The Copenhagen Psychosocial Questionnaire--a tool for the assessment and improvement of the psychosocial work environment. *Scand J Work Env Health* 2005;**31**: 438-49.
2. Pejtersen JH, Kristensen TS, Borg V, Bjorner JB. The second version of the Copenhagen Psychosocial Questionnaire. *Scand J Public Health* 2010;**38**: 8-24.
3. Burr H, Bjorner JB, Kristensen TS, Tüchsen F, Bach E. Trends in the Danish work environment in 1990–2000 and their associations with labor-force changes. *Scand J Work Env Health* 2003;**29**: 270-9.
4. Feveile H, Olsen O, Burr H, Bach E. Danish Work Environment Cohort Study 2005: From idea to sampling design. *Statistics in Transition* 2007;**8**: 441-58.
5. Kivimaki M, Lawlor DA, Smith GD, Kouvonen A, Virtanen M, Elovainio M, Vahtera J. Socioeconomic Position, Co-Occurrence of Behavior-Related Risk Factors, and Coronary Heart Disease: the Finnish Public Sector Study. *Am J Public Health* 2007;**97**: 874-9.
6. Korkeila K, Suominen S, Ahvenainen J, Ojanlatva A, Rautava P, Helenius H, Koskenvuo M. Non-response and related factors in a nation-wide health survey. *Eur J Epidemiol* 2001;**17**: 991-9.
7. Schmermund A, Mohlenkamp S, Stang A, Gronemeyer D, Seibel R, Hirche H, Mann K, Siffert W, Lauterbach K, Siegrist J, Jockel KH, Erbel R. Assessment of clinically silent atherosclerotic disease and established and novel risk factors for predicting myocardial infarction and cardiac death in healthy middle-aged subjects: rationale and design of the Heinz Nixdorf RECALL Study. Risk Factors, Evaluation of Coronary Calcium and Lifestyle. *Am Heart J* 2002;**144**: 212-8.
8. Stang A, Moebus S, Dragano N, Beck EM, Mohlenkamp S, Schmermund A, Siegrist J, Erbel R, Jockel KH. Baseline recruitment and analyses of nonresponse of the Heinz Nixdorf Recall Study: identifiability of phone numbers as the major determinant of response. *Eur J Epidemiol* 2005;**20**: 489-96.
9. Nielsen M, Kristensen T, Smith-Hansen L. The Intervention Project on Absence and Well-being (IPAW): design and results from the baseline of a 5-year study. *Work and Stress* 2002;**16**: 191-206.
10. Nielsen ML, Rugulies R, Christensen KB, Smith-Hansen L, Bjorner JB, Kristensen T. Impact of the psychosocial work environment on registered absence from work: a two-year longitudinal study using the IPAW cohort. *Work and Stress* 2004;**18**: 323-35.
11. de Groot W, Dekker R, The Dutch System of Official Social Surveys. Mannheim Centre for European Social Research, 2001.
12. Borritz M, Rugulies R, Bjorner JB, Villadsen E, Mikkelsen OA, Kristensen TS. Burnout among employees in human service work: design and baseline findings of the PUMA study. *Scand J Public Health* 2006;**34**: 49-58.
13. Marmot M, Brunner E. Cohort Profile: the Whitehall II study. *Int J Epidemiol* 2005;**34**: 251-6.
14. Peter R, Alfredsson L, Hammar N, Siegrist J, Theorell T, P. W. High effort, low reward, and cardiovascular risk factors in employed Swedish men and women: baseline results from the WOLF Study. *J Epidemiol Community Health* 1998;**52**: 540-7
15. Alfredsson L, Hammar N, Fransson E, de Faire U, Hallqvist J, Knutsson A, Nilsson T, Theorell T, Westerholm P. Job strain and major risk factors for coronary heart disease among employed males and females in a Swedish study on work, lipids and fibrinogen. *Scandi J Work Epidemiol Health* 2002;**28**: 238-48.

eAppendix 2. Details of exposures, outcomes and covariates

Ascertainment of exposures and outcomes

Weekly working hours were ascertained from baseline self-report questions on usual weekly working hours and defined as the total number of hours in the main job and any secondary jobs. Questions this information was based on are provided in Table S2.

Cancer events were identified from national cancer, hospitalisation and death registers in all studies apart from HNR. [1] In this study, cancer events ascertained from hospital records, interviews with the relevant participants' physicians or death records for the participants who had cancer recorded in the baseline medical examination or who reported having cancer during follow-up, and [2] Cancer cases were categorised according to the type and time of diagnosis of their first cancer. The date of the cancer event was defined as the date of diagnosis or hospital admission due to cancer, whichever came first. We excluded individuals whose first record of cancer came from their death certificate (n=10), as date of diagnosis for these cancers was uncertain. Codes for the incident cancer events were harmonised using ICD-10 (International Classification of Diseases, version 10) as any cancer (ICD-10 codes C00-C97), colorectal (C18-C20), lung (C34), female breast (C50) and prostate (C61) cancers.

Selection of covariates

Potential confounders were age, sex, socioeconomic position, shift work and night-time work. Age and sex are important (causal and otherwise) cancer risk factors [3] and many cancers are socioeconomically patterned. [4] In our data working long hours was more common in the middle age groups than among young workers or those nearing retirement age. We hypothesised that this pattern would be generalisable to other populations if, for example,

people in the middle age groups work long hours to meet the financial demands of a family, establish their careers or because they have reached a managerial-level position (where long hours can be a requirement). Young and old workers, on the other hand, may work part-time for educational or family reasons, or have taken partial retirement, respectively. Based on the distribution of working hours by sex and socioeconomic position in our data, we also hypothesised that the propensity to work long hours might more generally differ between men and women (although this could relate to the gender distribution of particular jobs or types of work). For those in a low socioeconomic position, this could be due to a need to incur extra income by working overtime or lack of authority over the hours they work. For those at the high end of the socioeconomic spectrum working long hours could be due to responsibility for and authority over other people's work, which are typical of managerial-level positions. There is weak epidemiological evidence of night-time or shift work being associated with an increased risk of breast cancer in women and possibly some cancers in men. [5-9] The proposed mechanisms for these associations involve night-time and shift work potentially disrupting the body's circadian rhythm, altering the nocturnal production of melatonin and other hormones (which could impact particularly on the development of hormone-related cancers, such as many breast cancers), possibly leading to sleep deprivation (which can impair immune system functions) and disrupting healthy lifestyles (leading to, for example, obesity). [10, 11]

Potential mediators were tobacco smoking, alcohol intake and body mass index (BMI). These lifestyle-related factors are associated with the risk of many cancers [12-16] and we hypothesised that they could be on the causal pathway between long working hours and cancer if, for instance, working long hours (or stress or lack of time for outside work activities associated with working long hours) leads workers to eat less healthily, smoke more or drink more alcohol. [17-19] The likely associations with site-specific cancers vary. For example, shift work or night

work may be related to breast cancer only, smoking particularly to lung, colorectal and possibly prostate cancers, alcohol intake to colorectal and breast cancers (but not lung cancer) and obesity to colorectal, menopausal breast and aggressive prostate cancers.[20, 21]

Ascertainment of covariates

Age and sex were ascertained from population or employers' registries or interview (COPSOQ-I and II, DWECS, FPS, HNR, IPAW, PUMA, WOLF Norrland and WOLF Stockholm) or self-report questionnaires (in HeSSup, POLS and Whitehall II). Data on occupation-based socioeconomic position were obtained from employers' or other registers (in COPSOQ-I and II, DWECS, FPS, IPAW, PUMA) or self-report questionnaires (in HNR, POLS, Whitehall II, WOLF Norrland and WOLF Stockholm). In HeSSup, socioeconomic position was defined based on self-reported highest educational qualification. BMI (weight in kilograms divided by height in metres squared) was calculated from height and weight, which were self-reported (in DWECS, FPS, HeSSup, HNR, IPAW, POLS and PUMA) or measured at baseline examination (in Whitehall II, WOLF Norrland and WOLF Stockholm). Information on shift work, night time work, weekly alcohol intake and smoking were obtained from self-report questionnaires in all studies. All covariates were measured at study baseline and harmonised across the studies as reported previously. [18, 22-24]

Table S2. Questions used to obtain information on weekly working hours

Study	Country	Question(s) on working hours<br>Answer fields
Work, Lipids and Fibrinogen (WOLF) Stockholm	Sweden	How many hours do you normally work per week, including overtime, and how are these hours distributed on average?<br>Day work (06:00–18:00 hours), number of hours<br>Evening work (18:00–22:00 hours), number of hours<br>Night work (22:00–06:00 hours), number of hours
Whitehall II	United Kingdom	On an average weekday, approximately how many hours do you spend on the following activities –<br>Work (daytime and work brought home)?
Work, Lipids and Fibrinogen (WOLF) Norrland	Sweden	How many hours do you normally work per week, including overtime, and how are these hours distributed on average?<br>Day work(06:00–18:00 hours), number of hours<br>Evening work (18:00–22:00 hours), number of hours<br>Night work (22:00–06:00 hours), number of hours
Intervention Project on Absence and Well-being (IPAW)	Denmark	How many hours do you usually work, on average, per week?<br>Number of hours
Copenhagen Psychosocial Questionnaire I (COPSOQ-I)	Denmark	How many hours do you usually work per week?<br>Number of hours in main job<br>Number of hours in any secondary job
Health and Social Support study (HeSSup)	Finland	Estimate the number of weekly hours you work in your main job<br>Number of hours<br>Number of minutes<br>Estimate the number of weekly hours you work in your secondary job<br>Number of hours<br>Number of minutes
Burnout, Motivation and Job Satisfaction study (Danish acronym PUMA)	Denmark	How many hours do you usually work per week?<br>Number of hours in main job<br>Number of hours in any secondary job
Danish Work Environment Cohort Study (DWECS)	Denmark	How many hours do you normally work in your main job, including any scheduled hours, paid overtime and other extra work, such as working at home?<br>Hours on average<br>How many hours do you normally work in your secondary job, including any scheduled hours, paid overtime and other extra work, such as working at home?<br>Hours on average
Finnish Public Sector study (FPS)	Finland	How many contracted hours do you work per week?<br>How many hours of extra and/or overtime work do you do per week?
Heinz-Nixdorf Recall study (HNR)	Germany	How many hours, on average, is your weekly working time (including any regular overtime work)?
Permanent Onderzoek Leefsituatie (POLS)	Netherlands	How many hours do you work on a normal week (not including any unpaid work)?
Copenhagen Psychosocial Questionnaire II (COPSOQ-II)	Denmark	How many hours do you actually work per week (including overtime and any secondary jobs)?<br>Number of hours per week

References

1. Heikkila K, Nyberg ST, Theorell T, Fransson EI, Alfredsson L, Bjorner JB, Bonenfant S, Borritz M, Bouillon K, Burr H, Dragano N, Geuskens GA, et al. Work Stress and Cancer Risk: A Meta-analysis of 5 700 Incident Cancer Events in 116 000 European Men and Women. *Br Med J* 2013;**346**: f165.
2. Bokhof B, Eisele L, Erbel R, Moebus S, Investi HNRS. Agreement between different survey instruments to assess incident and prevalent tumors and medical records - results of the Heinz Nixdorf Recall Study. *Cancer Epidemiol* 2014;**38**: 181-92.
3. Weinberg RA. *The Biology of Cancer*ed.: Garland Science, Taylor and Francis Group, LLC, 2007.
4. Social inequalities and cancer. *IARC scientific publications* 1997: 1-15.
5. Ijaz S, Verbeek J, Seidler A, Lindbohm ML, Ojajarvi A, Orsini N, Costa G, Neuvonen K. Night-shift work and breast cancer--a systematic review and meta-analysis. *Scand J Work Env Health* 2013;**39**: 431-47.
6. Jia Y, Lu Y, Wu K, Lin Q, Shen W, Zhu M, Huang S, Chen J. Does night work increase the risk of breast cancer? A systematic review and meta-analysis of epidemiological studies. *Cancer Epidemiol* 2013;**37**: 197-206.
7. Kamdar BB, Tergas AI, Mateen FJ, Bhayani NH, Oh J. Night-shift work and risk of breast cancer: a systematic review and meta-analysis. *Breast Cancer Res Treat* 2013;**138**: 291-301.
8. Parent M-E, El-Zein M, Rousseau M-C, Pintos J, Siemiatycki J. Night work and the risk of cancer among men. *Am J Epidemiol* 2012;**176**: 751-9.
9. Wang F, Yeung KL, Chan WC, Kwok CC, Leung SL, Wu C, Chan EY, Yu IT, Yang XR, Tse LA. A meta-analysis on dose-response relationship between night shift work and the risk of breast cancer. *Ann Oncol* 2013;**24**: 2724-32.
10. Davis S, Mirick DK. Circadian disruption, shift work and the risk of cancer: a summary of the evidence and studies in Seattle. *Cancer Causes Control* 2006;**17**: 539-45.
11. Haus EL, Smolensky MH. Shift work and cancer risk: potential mechanistic roles of circadian disruption, light at night, and sleep deprivation. *Sleep Med Rev* 2013;**17**: 273-84.
12. Allen NE, Beral V, Casabonne D, Kan SW, Reeves GK, Brown A, Green J. Moderate alcohol intake and cancer incidence in women. *J Natl Cancer Inst* 2009;**101**: 296-305.
13. Botteri E, Iodice S, Bagnardi V, Raimondi S, Lowenfels AB, Maisonneuve P. Smoking and colorectal cancer: a meta-analysis. *JAMA* 2008;**300**: 2765-78.
14. Fedirko V, Tramacere I, Bagnardi V, Rota M, Scotti L, Islami F, Negri E, Straif K, Romieu I, La Vecchia C, Boffetta P, Jenab M. Alcohol drinking and colorectal cancer risk: an overall and dose-response meta-analysis of published studies. *Ann Oncol* 2011;**22**: 1958-72.
15. Freedman ND, Leitzmann MF, Hollenbeck AR, Schatzkin A, Abnet CC. Cigarette smoking and subsequent risk of lung cancer in men and women: analysis of a prospective cohort study. *Lancet Oncol* 2008;**9**: 649-56.
16. Huncharek M, Haddock KS, Reid R, Kupelnick B. Smoking as a risk factor for prostate cancer: a meta-analysis of 24 prospective cohort studies. *Am J Public Health* 2010;**100**: 693-701.
17. Virtanen M, Jokela M, Nyberg S, Madsen I, Lallukka T, Ahola K, Alfredsson L, Batty G, Bjorner J, Borritz M, Burr H, Casini A, et al. Long working hours and alcohol use: systematic review and meta-analysis of published studies and unpublished individual participant data. *Br Med J* 2015;**350:g7772**
18. Heikkilä K, Nyberg ST, Fransson EI, Alfredsson L, De Bacquer D, Bjorner JB, Bonenfant S, Borritz M, Burr H, Clays E, Casini A, Dragano N, et al. Job Strain and Tobacco Smoking: An Individual-participant Data Meta-analysis of 166 130 Adults in 15 European Studies. *PLoS ONE* 2012;**7**: e35463.
19. Escoto KH, Laska MN, Larson N, Neumark-Sztainer D, Hannan PJ. Work hours and perceived time barriers to healthful eating among young adults. *American journal of health behavior* 2012;**36**: 786-96.

20. Humans IWGotEoCRt. Personal habits and indoor combustions. Volume 100 E. A review of human carcinogens. *IARC Monographs on the Evaluation of Carcinogenic Risks to Humans / World Health Organization, International Agency for Research on Cancer* 2012;**100**: 1-538.
21. Straif K, Baan R, Grosse Y, Secretan B, El Ghissassi F, Bouvard V, Altieri A, Benbrahim-Tallaa L, Cogliano V. Carcinogenicity of shift-work, painting, and fire-fighting. *Lancet Oncol* 2007;**8**: 1065-6.
22. Heikkila K, Nyberg ST, Fransson EI, Alfredsson L, De Bacquer D, Bjorner JB, Bonenfant S, Borritz M, Burr H, Clays E, Casini A, Dragano N, et al. Job Strain and Alcohol Intake: A Collaborative Meta-analysis of Individual-participant Data from 140 000 Men and Women. *PLoS ONE* 2012;**7**: e40101.
23. Nyberg ST, Heikkila K, Fransson EI, Alfredsson L, De Bacquer D, Bjorner JB, Bonenfant S, Borritz M, Burr H, Casini A, Clays E, Dragano N, et al. Job strain in relation to body mass index: pooled analysis of 160 000 adults from 13 cohort studies. *J Int Med* 2012;**272**: 65-73.
24. Nyberg ST, Fransson EI, Heikkila K, Ahola K, Alfredsson L, Bjorner JB, Borritz M, Burr H, Dragano N, Goldberg M, Hamer M, Jokela M, et al. Job Strain as a Risk Factor for Type 2 Diabetes: A Pooled Analysis of 124,808 Men and Women. *Diabetes Care* 2014;**37** 2268-75

eAppendix 3. Associations of weekly working hours with overall incident cancer

Figure S1. Working hours and any incident cancer (adjusted for age and sex only)

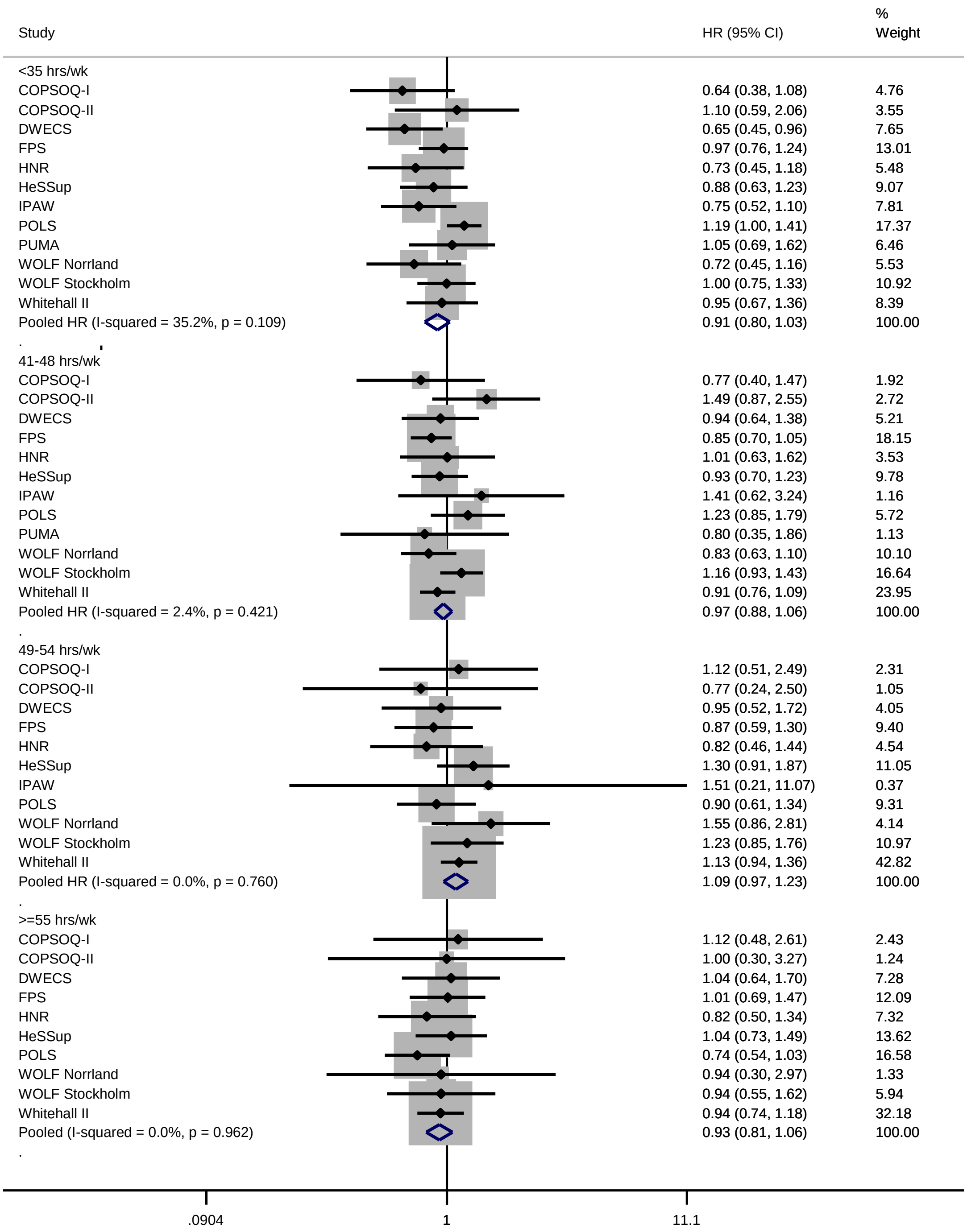

Figure S2. Working hours and any incident cancer (adjusted for age, sex, socioeconomic position, shift work and night-time work)

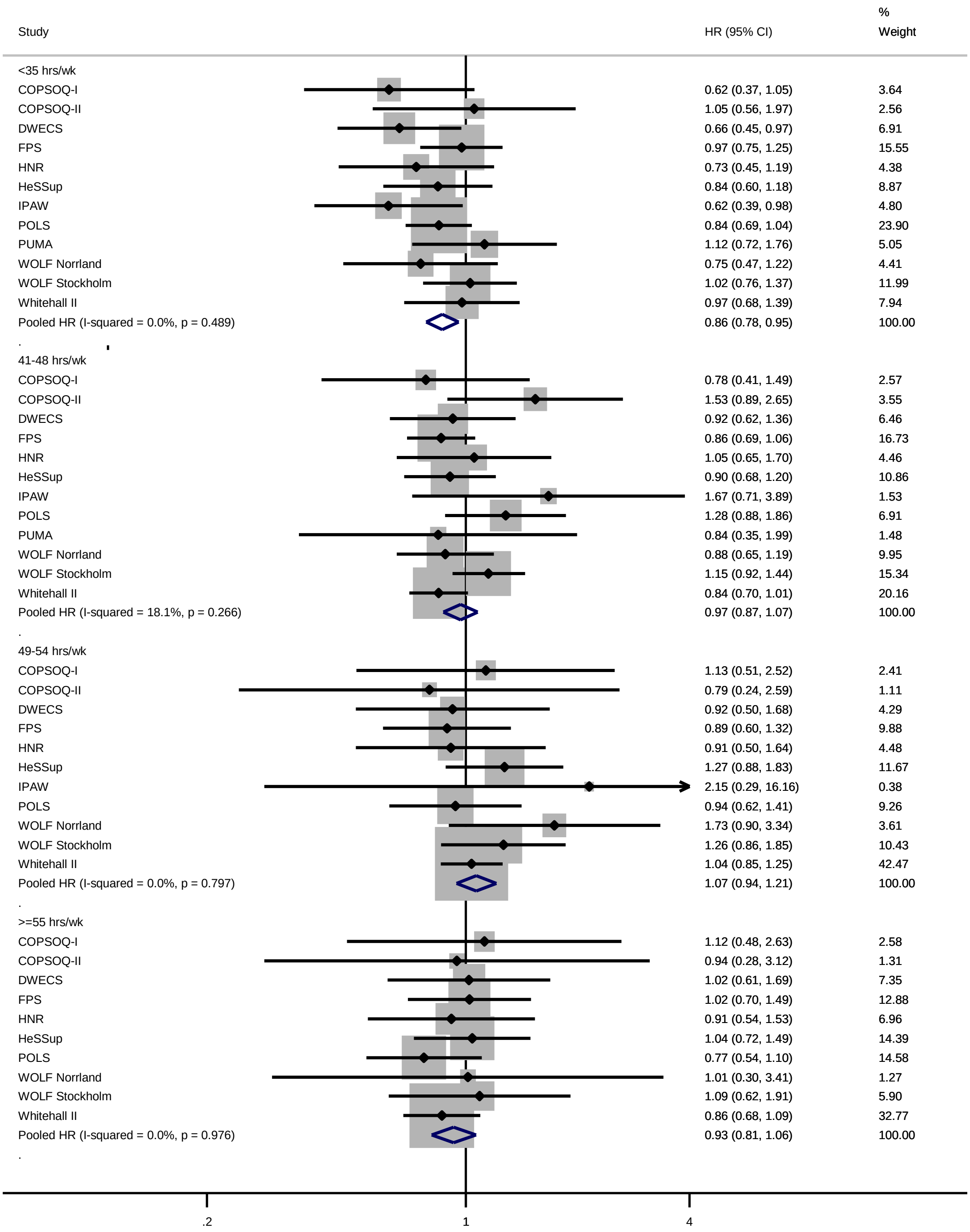

Figure S3. Working hours and any incident cancer (adjusted for age, sex, socioeconomic position, shift work, night-time work, BMI, smoking and alcohol intake)

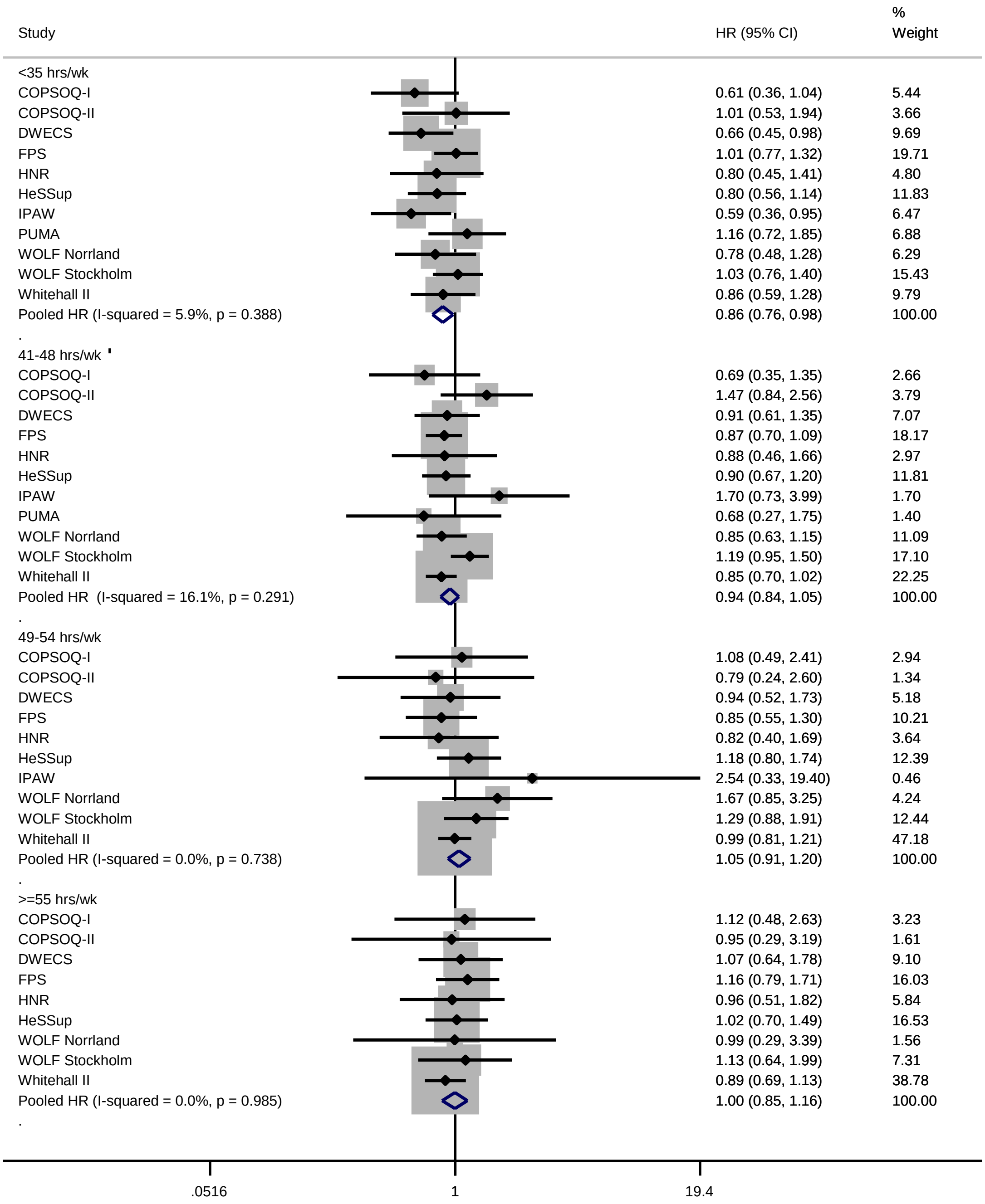

eAppendix 4. Associations of weekly working hours with incident colorectal cancer

Figure S4. Working hours and incident colorectal cancer (adjusted for age and sex only)

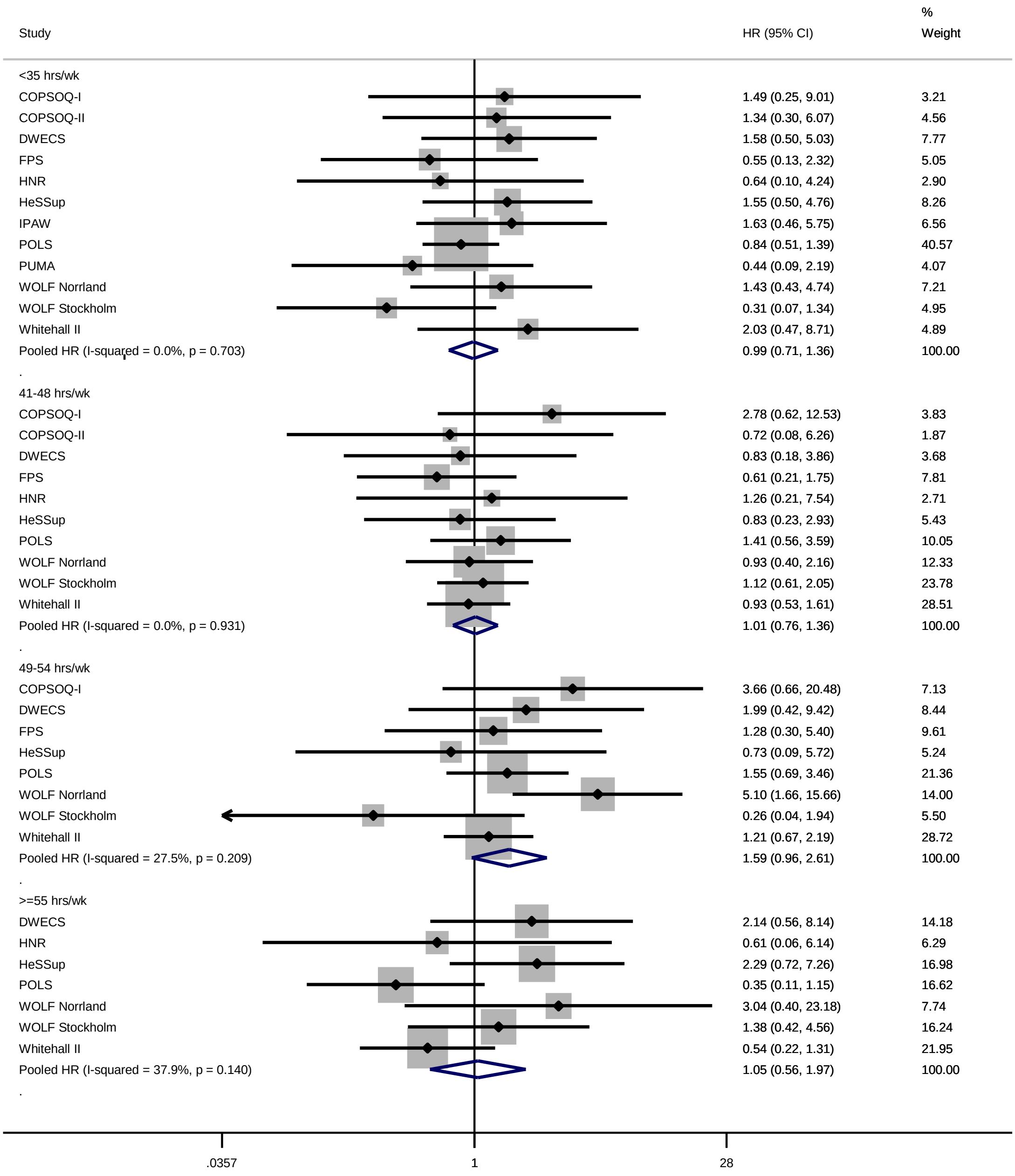

Figure S5. Working hours and incident colorectal cancer (adjusted for age, sex, socioeconomic position, shift work and night-time work)

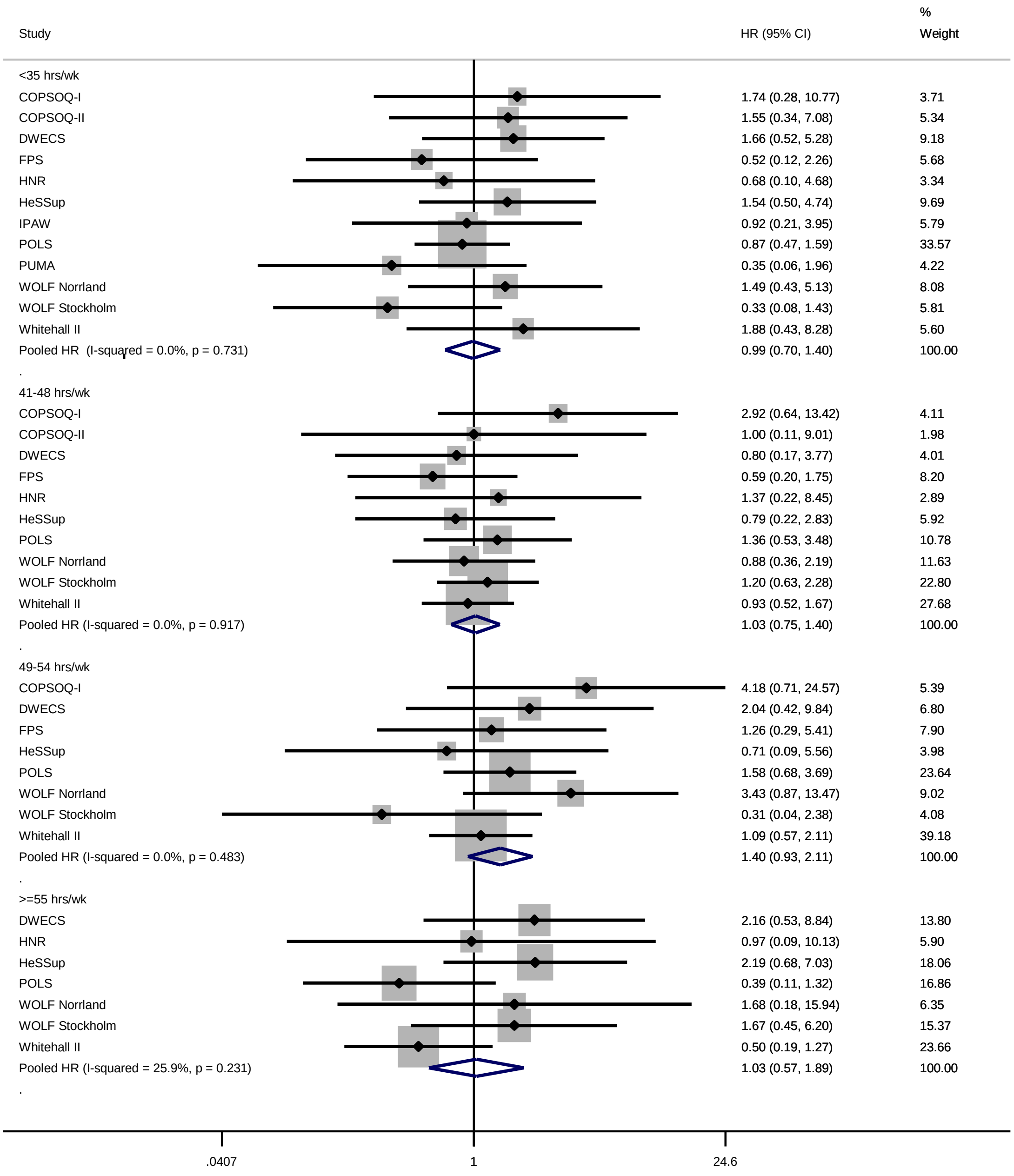

Figure S6. Working hours and incident colorectal cancer (adjusted for age, sex, socioeconomic position, shift work, night-time work, BMI, smoking and alcohol intake)

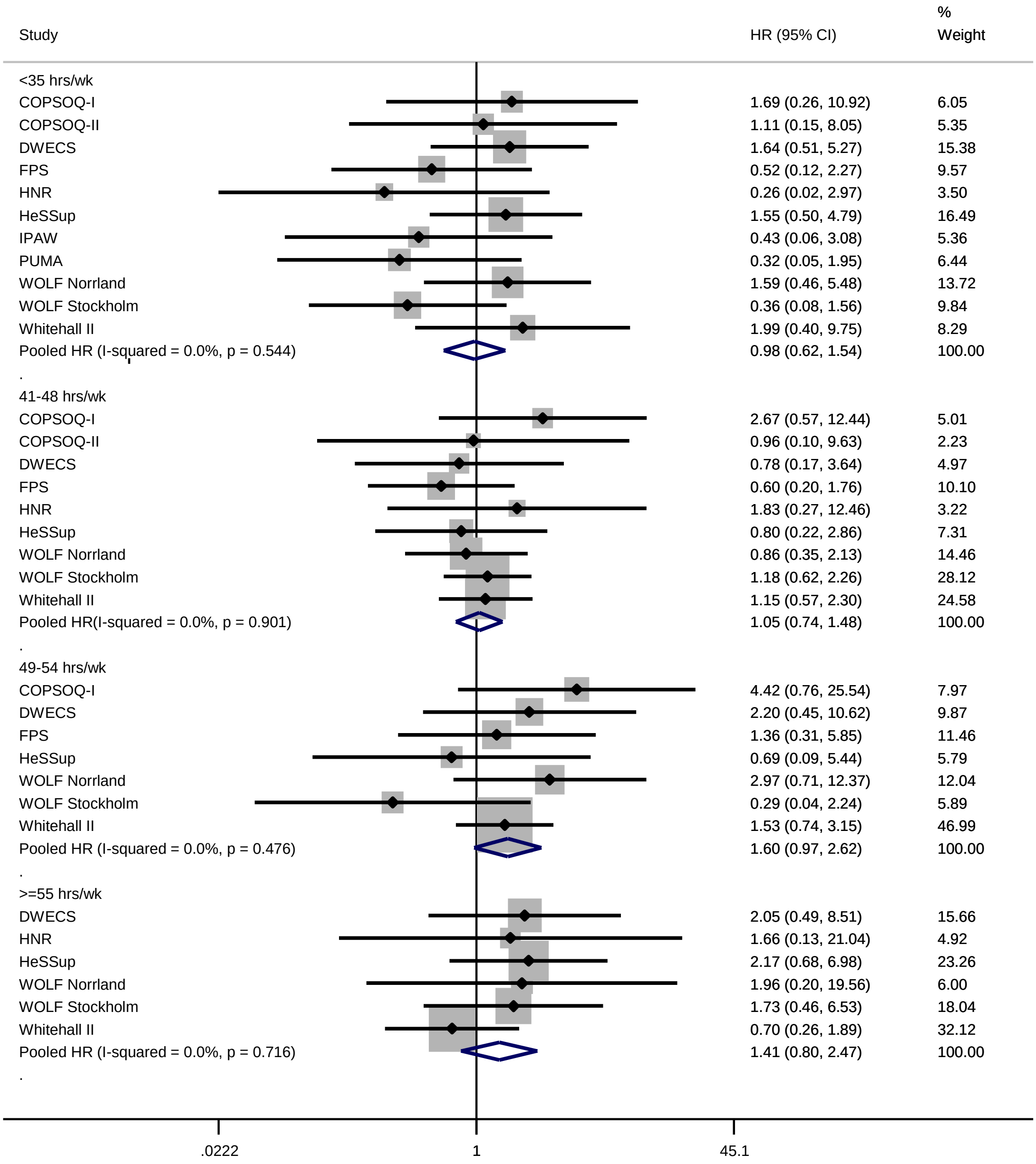

eAppendix 5. Associations of weekly working hours with incident lung cancer

Figure S7. Working hours and incident lung cancer (adjusted for age and sex only)

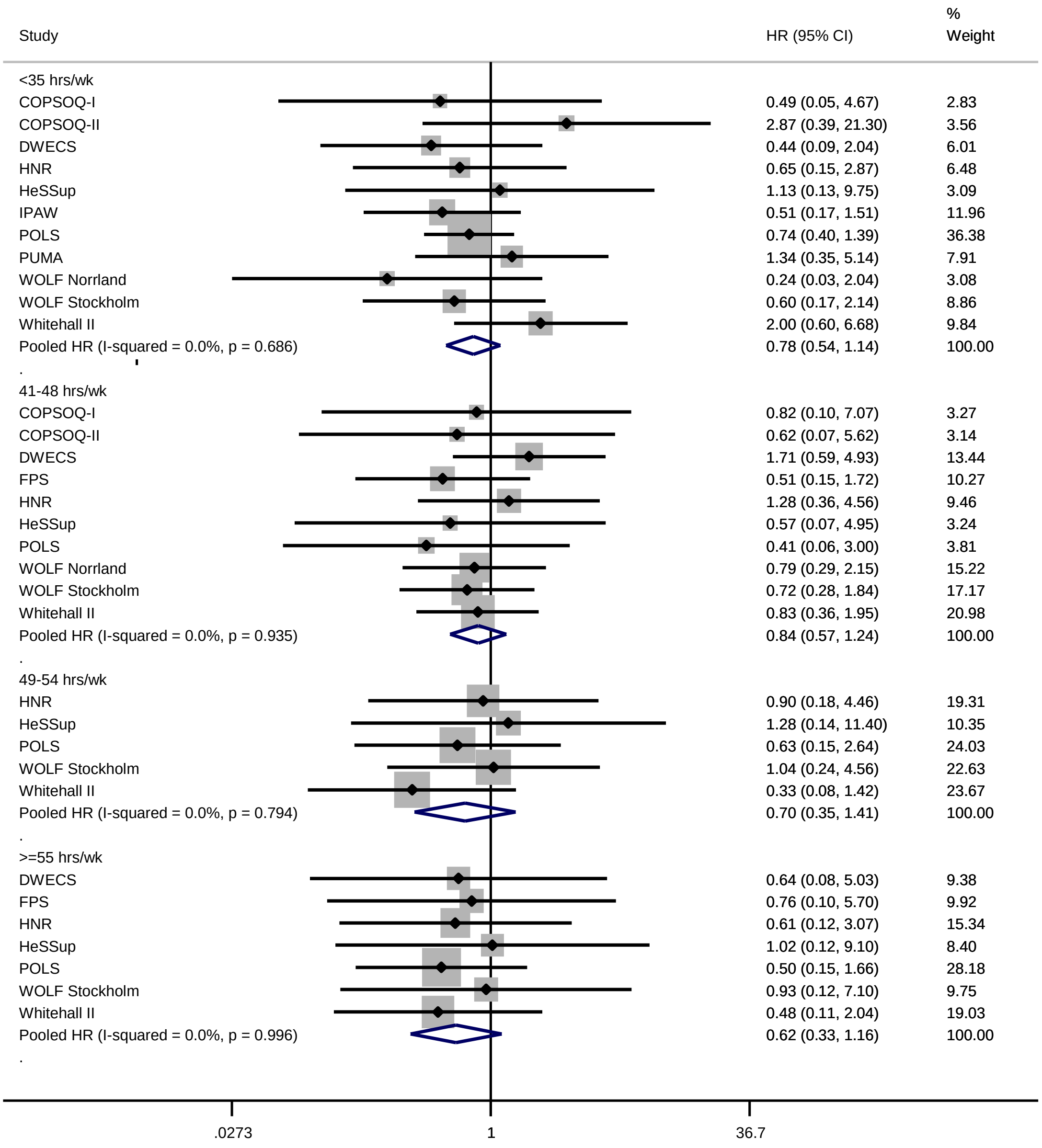

Figure S8. Working hours and incident lung cancer (adjusted for age, sex, socioeconomic position, shift work and night-time work)

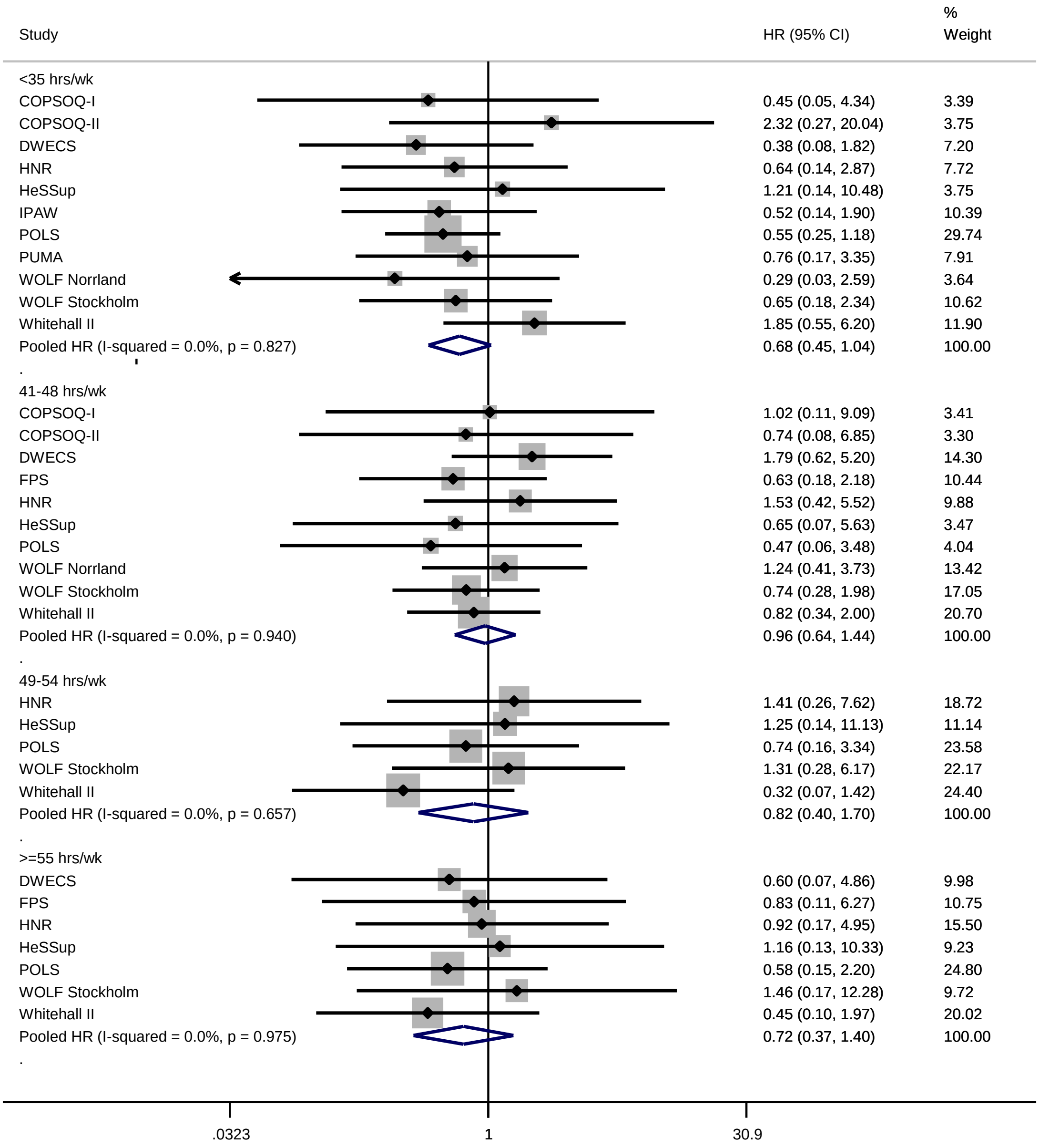

Figure S9. Working hours and incident lung cancer (adjusted for age, sex, socioeconomic position, shift work, night-time work, BMI, smoking and alcohol intake)

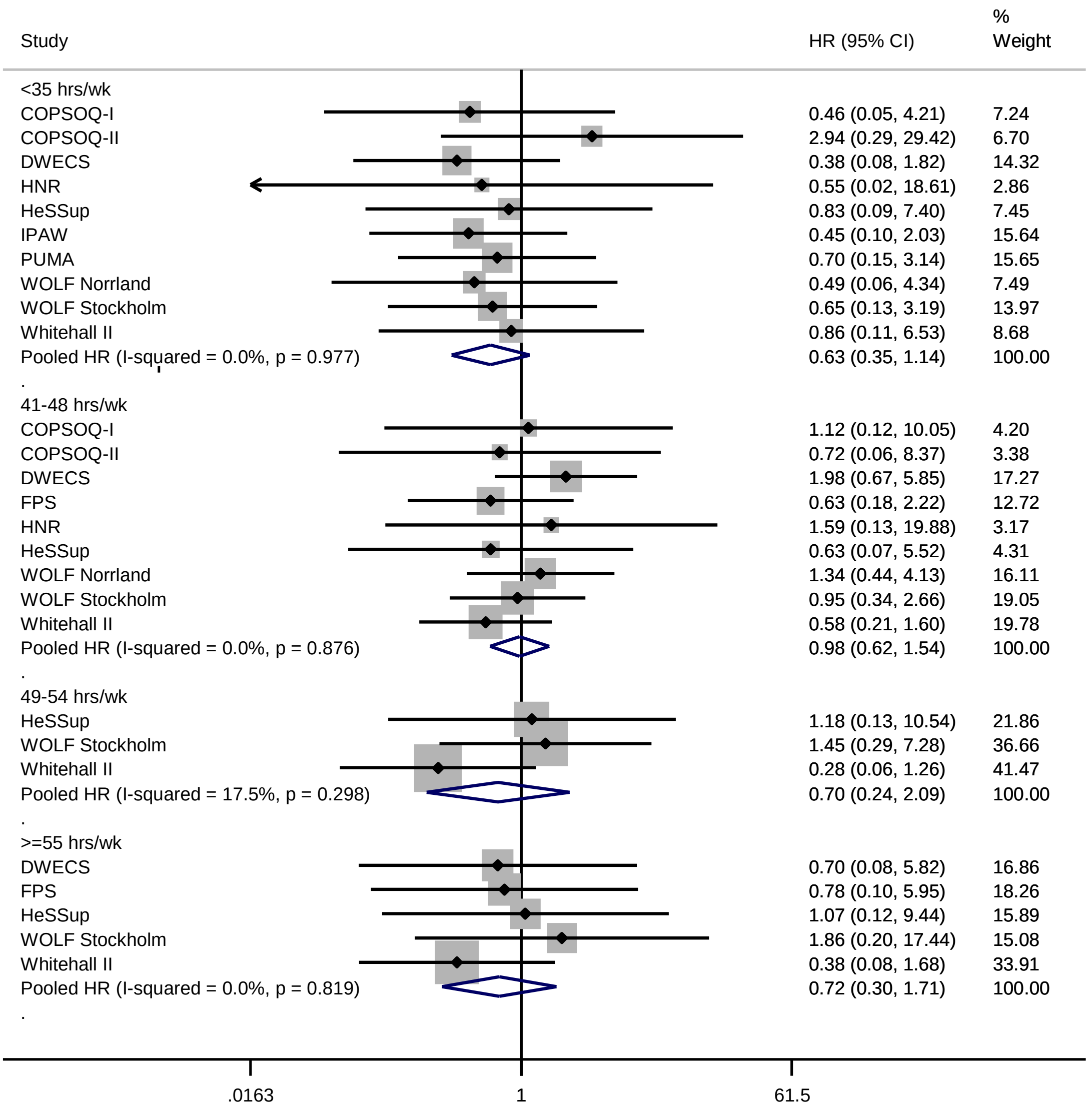

eAppendix 6. Associations of weekly working hours with incident breast cancer

Figure S10. Working hours and incident breast cancer (adjusted for age only)

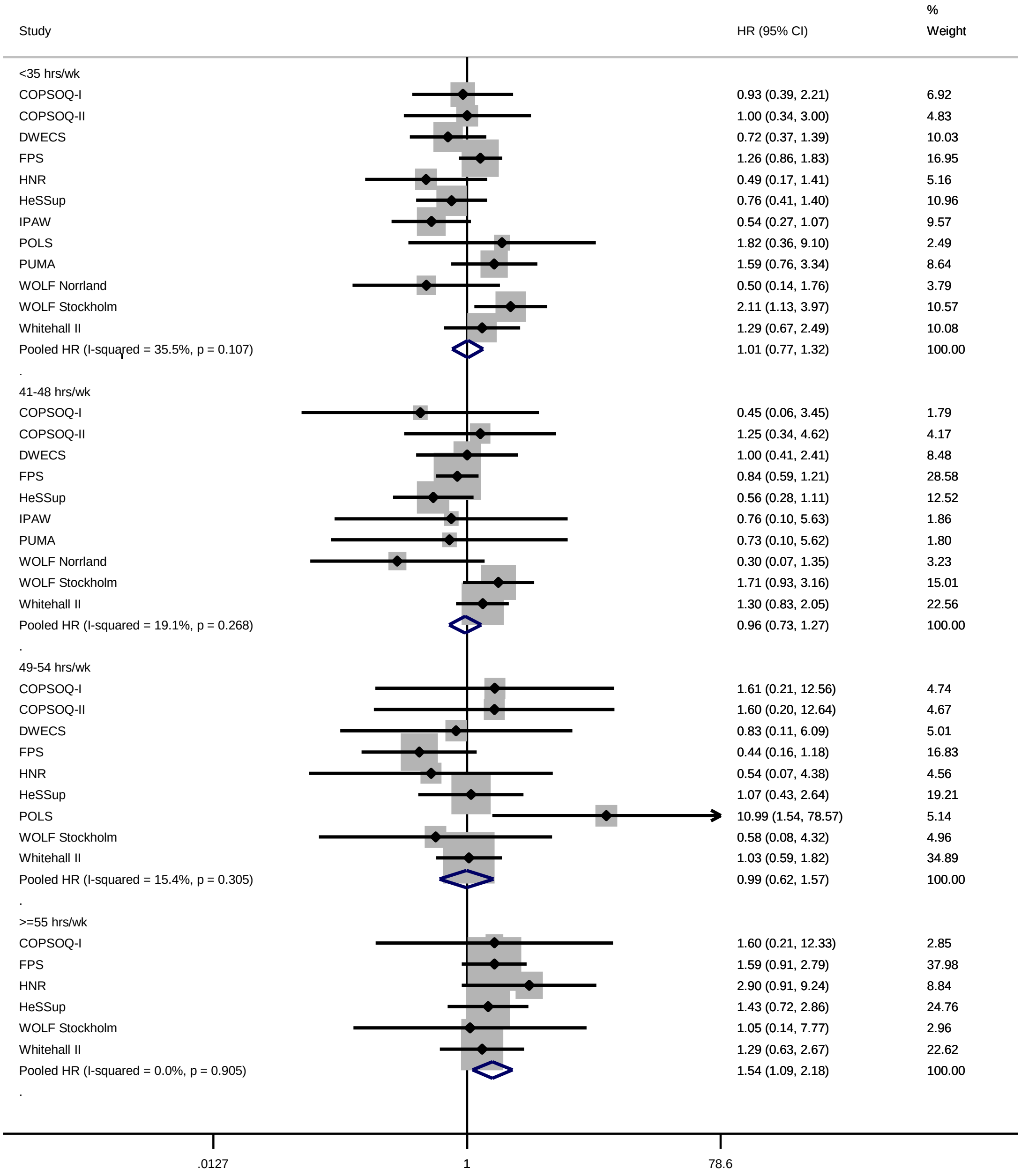

Figure S11. Working hours and incident breast cancer (adjusted for age, socioeconomic position, shift work and night-time work)

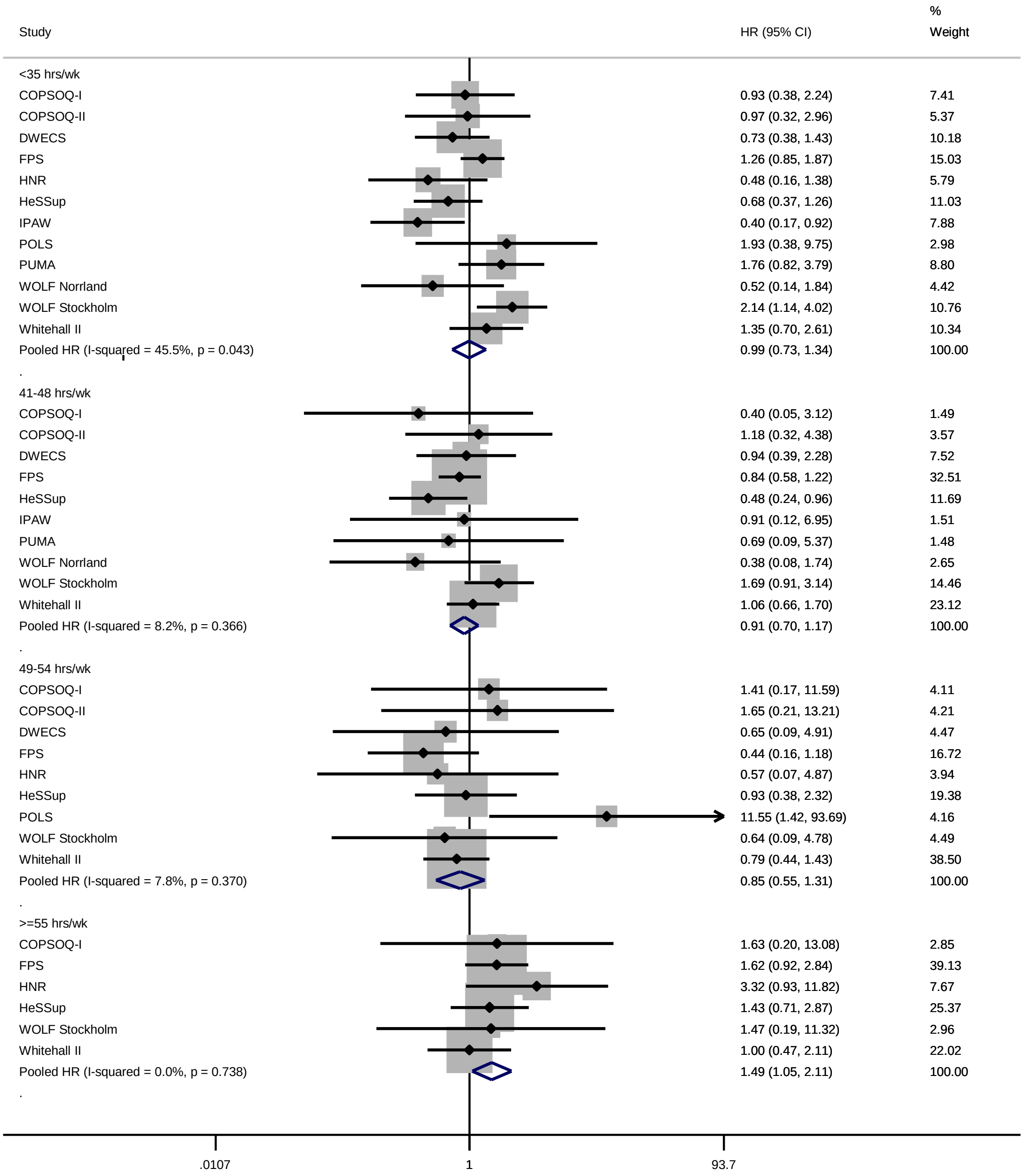

Figure S12. Working hours and incident breast cancer (adjusted for age, socioeconomic position, shift work, night-time work, BMI, smoking and alcohol intake)

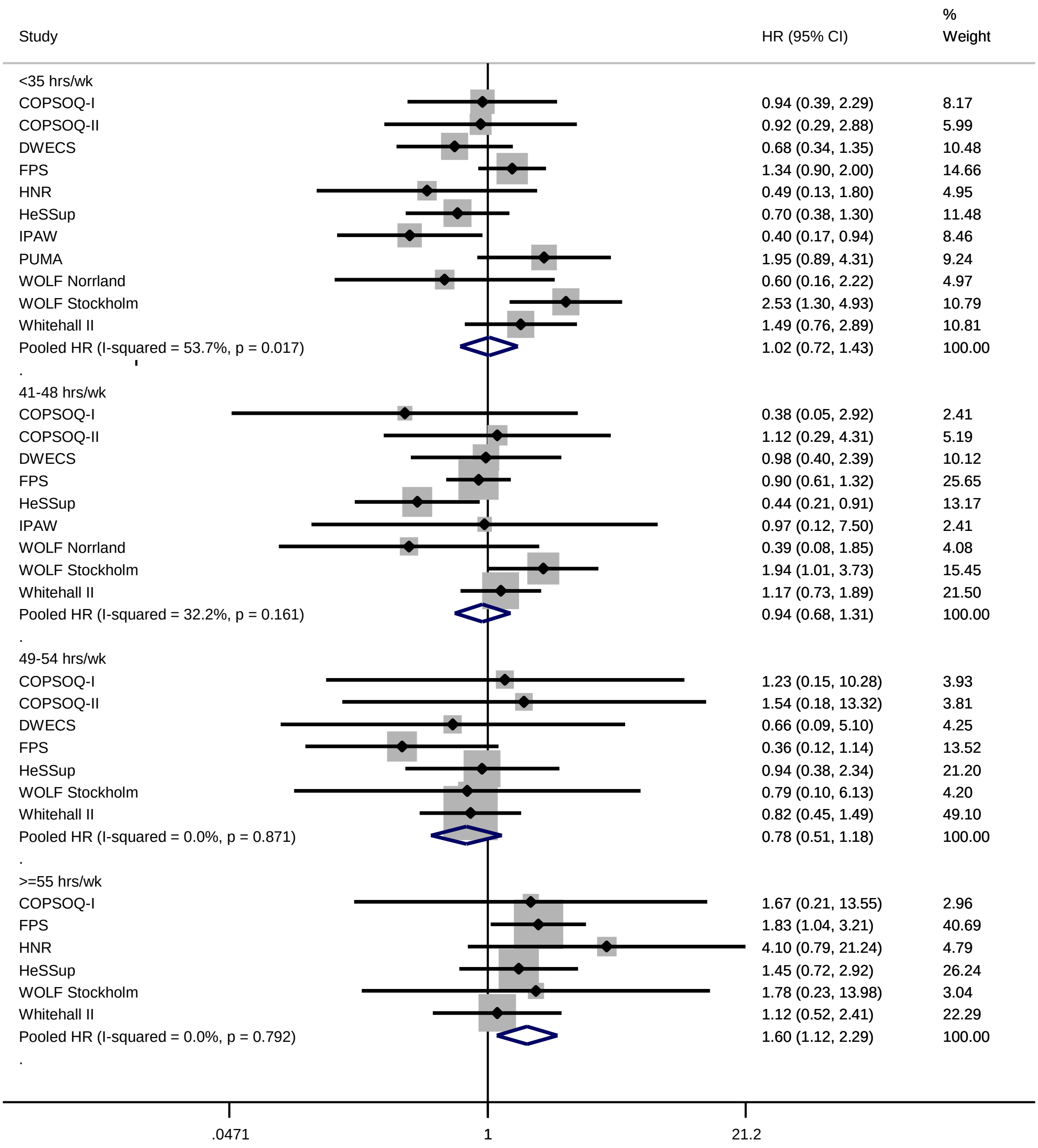

eAppendix 7. Associations of weekly working hours with incident prostate cancer

Figure S13. Working hours and incident prostate cancer (adjusted for age only)

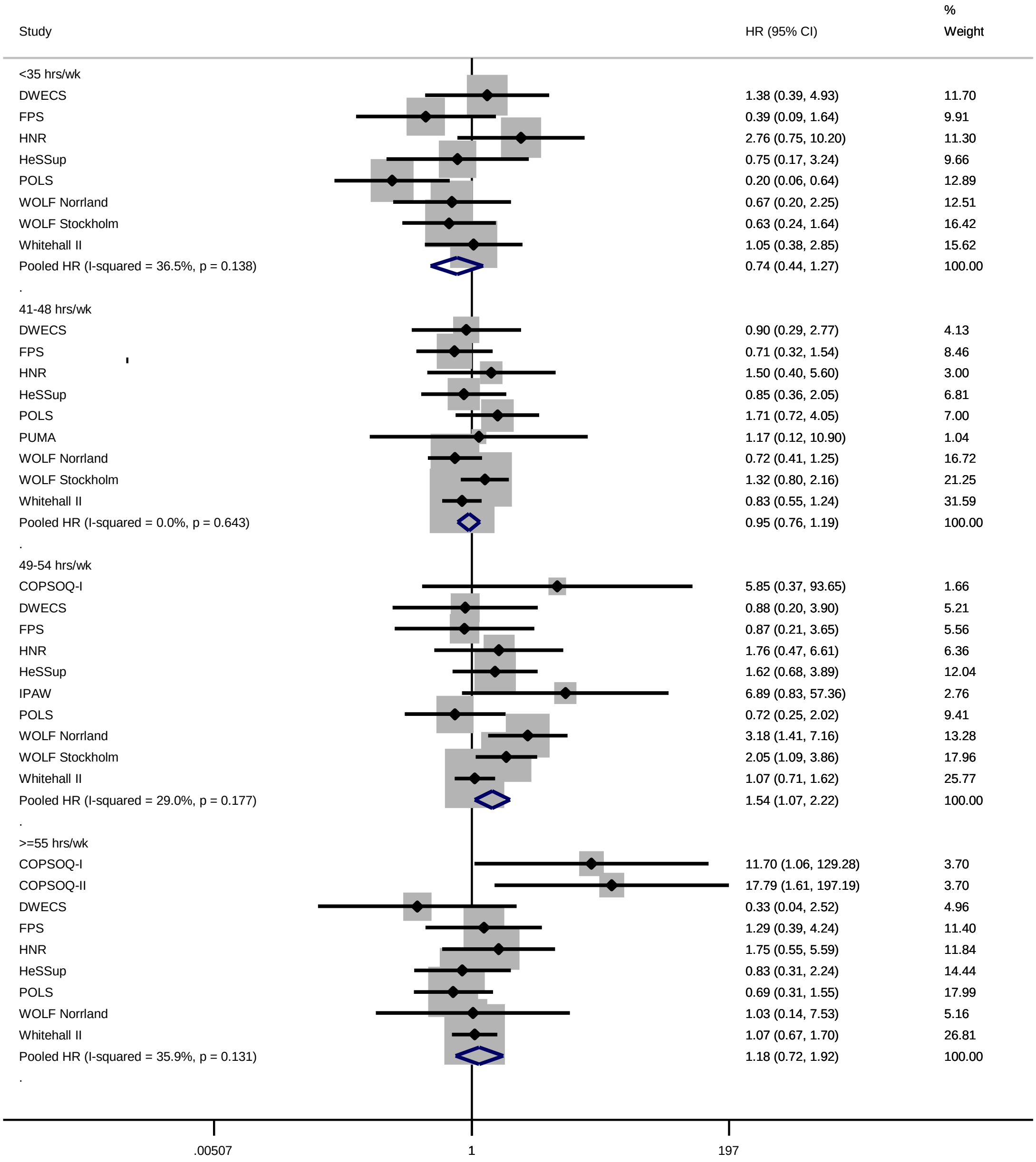

Figure S14. Working hours and incident prostate cancer (adjusted for age, socioeconomic position, shift work and night-time work)

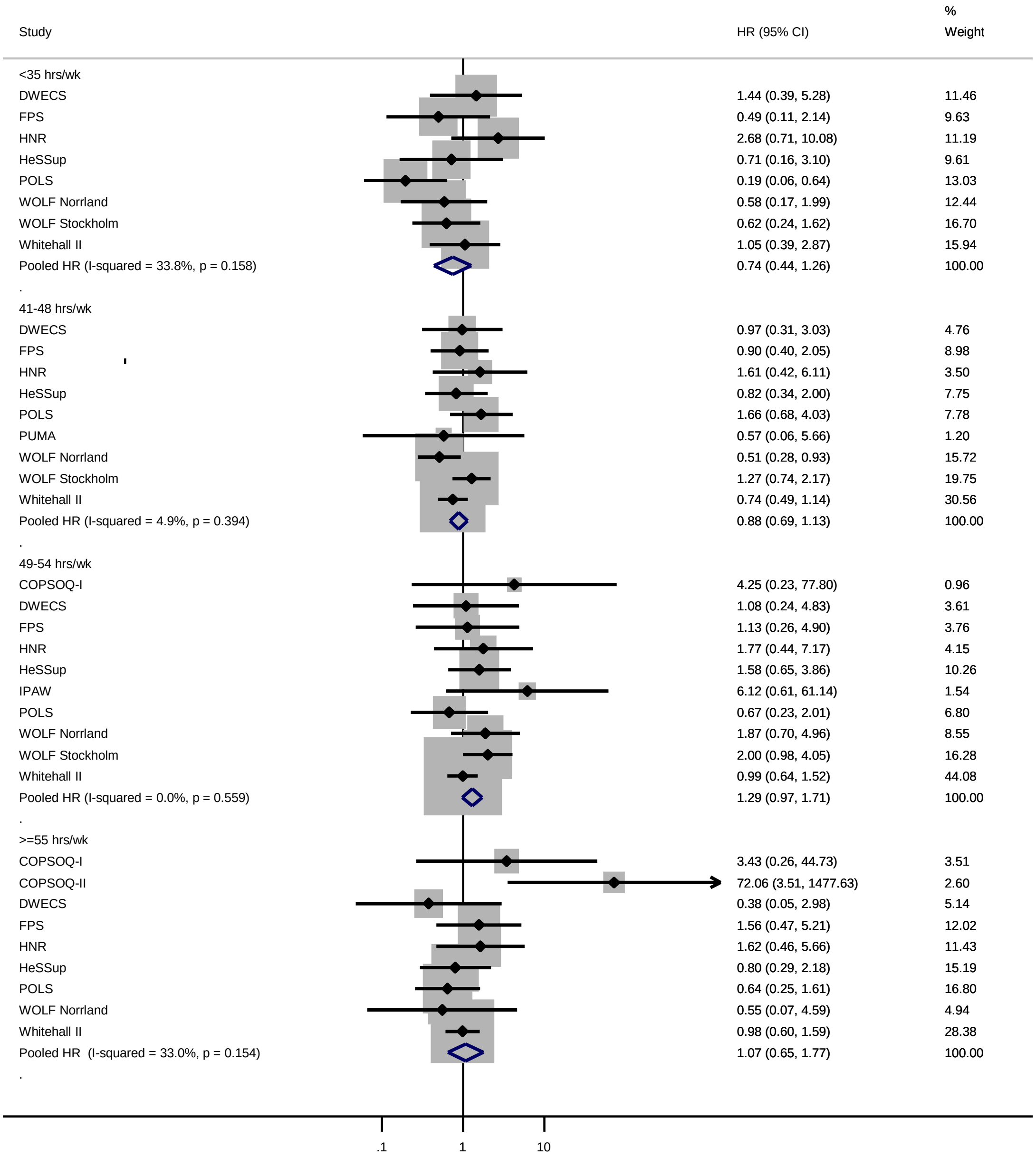

Figure S15. Working hours and incident prostate cancer (adjusted for age, socioeconomic position, shift work, night-time work, BMI, smoking and alcohol intakte)

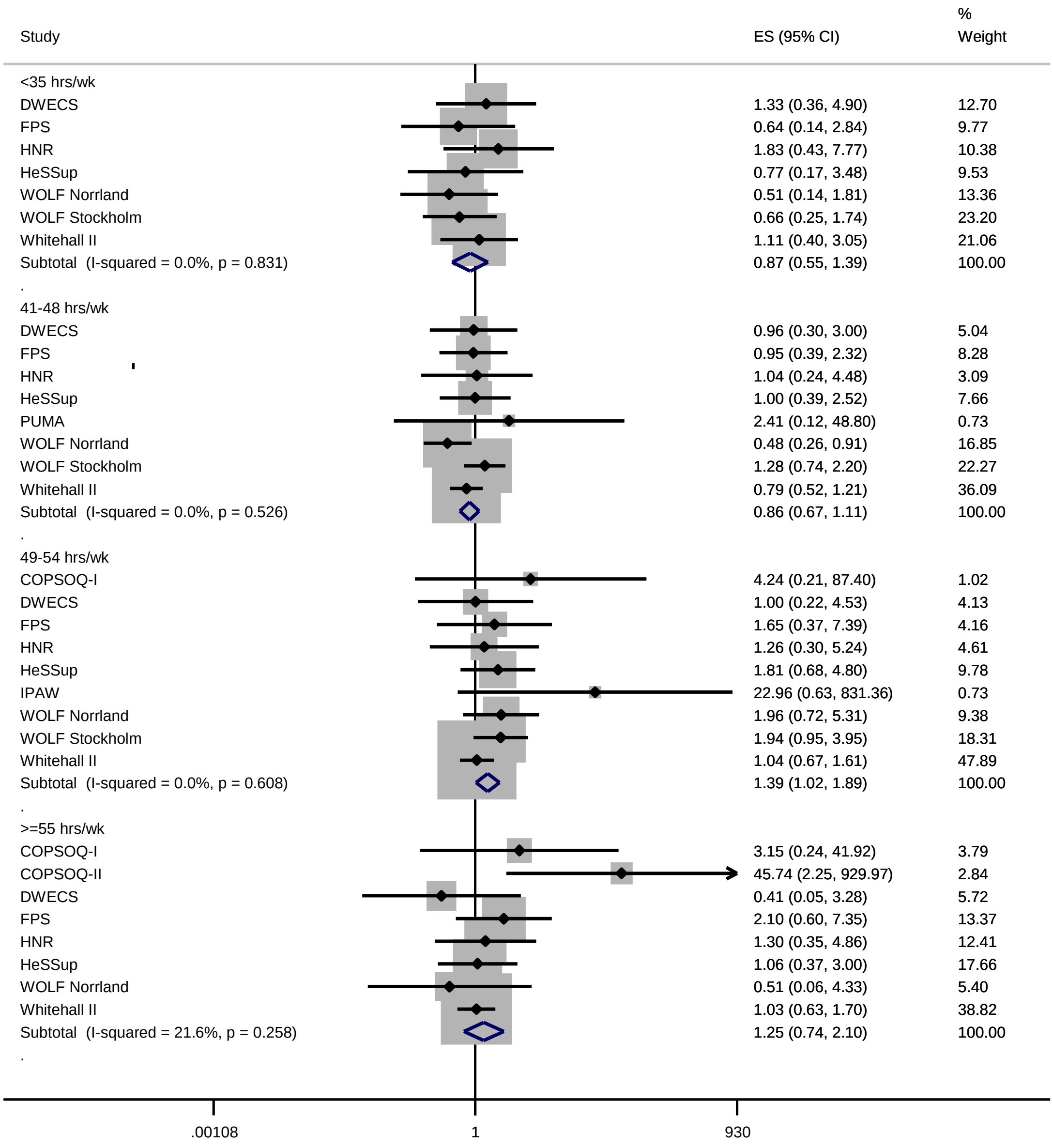

Supplement: Supplementary Information [file bjc20169x1.pdf]
